# Cytoglobin Promotes Cardiac Progenitor Cell Survival against Oxidative Stress via the Upregulation of the NFκB/iNOS Signal Pathway and Nitric Oxide Production

**DOI:** 10.1038/s41598-017-11342-6

**Published:** 2017-09-07

**Authors:** Shuning Zhang, Xiuchun Li, Frances L. Jourd’heuil, Shunlin Qu, Neil Devejian, Edward Bennett, David Jourd’heuil, Chuanxi Cai

**Affiliations:** 1 Center for Cardiovascular Sciences, Department of Molecular and Cellular Physiology, & Department of Medicine, Albany Medical College, Albany, NY 12208 USA; 2 Division of Pediatric Cardiothoracic Surgery, Albany Medical Center, Albany, NY 12208 USA; 3 Division of Cardiothoracic Surgery, Albany Medical Center, Albany, NY 12208 USA

## Abstract

Human cardiac stem/progenitor cells (hCPCs) may serve in regenerative medicine to repair the infarcted heart. However, this approach is severely limited by the poor survival of donor cells. Recent studies suggest that the mammalian globin cytoglobin (CYGB) regulates nitric oxide (NO) metabolism and cell death. In the present study, we found that CYGB is expressed in hCPCs. Through molecular approaches aimed at increasing or decreasing CYGB expression in hCPCs, we found that CYGB functions as a pro-survival factor in response to oxidative stress. This was associated with the upregulation of primary antioxidant systems such as peroxiredoxins-1, heme oxygenase-1, and anti-apoptotic factors, including BCL2, BCL-XL, and MCL1. Most significantly, we established that CYGB increased the expression of NFкB-dependent genes including iNOS, and that iNOS-dependent NO production was required for a feedforward loop that maintains CYGB expression. Our study delineates for the first time a role for a globin in regulating hCPC survival and establishes mechanistic insights in the function of CYGB. It provides a rationale for the exploration of the CYGB pathway as a molecular target that can be used to enhance the effectiveness of cardiac stem/progenitor cell therapy for ischemic heart disease.

## Introduction

Delineating new therapeutic interventions for ischemic heart disease has been extremely challenging. Pre-clinical and more recent clinical studies suggest that stem/progenitor cell based therapies may offer great potential for repairing the damaged heart after ischemia/reperfusion injury^1–5^. The heart harbors its own pool of c-kit positive resident progenitor cells, which are capable of differentiating into multiple cell types in the heart^1, 6^. Disappointingly, clinical reports also suggest that the majority of the progenitor cell approaches tested thus far exhibit only marginal, transient, or even negative effects^7, 8^. One of the major challenges associated with cell transplantation is the fact that the majority of the donor cells that manage to stay in the heart do not survive^9, 10^. Several methods to improve cell survival have been described, including heat shock of the cells prior to transplantation, forced expression of survival factors in the transplanted cells, and exposure of the transplant to soluble pro-survival factors^11, 12^. Although these interventions do seem to facilitate cell survival, there is clearly room for additional improvement.

The function of mammalian globins such as hemoglobin in erythrocytes and myoglobin in striated muscles are classically associated with oxygen transport^13–15^. Over the past fifteen years, novel globins that include androglobin, neuroglobin, and cytoglobin have been discovered^16–21^. Although they all bind molecular oxygen, their relatively low intracellular concentrations preclude any significant roles as oxygen transport or storage system. Significantly, cytoglobin (CYGB) is a hexacoordinated globin that is co-expressed with myoglobin in adult cardiomyocytes and upregulated during hypoxic events^21–23^. The role of CYGB in the heart is unknown but recent studies would indicate cytoprotective functions, potentially associated with direct or indirect antioxidant properties^24–27^. CYGB might also contribute to cell-mediated nitric oxide (NO) metabolism through NO dioxygenation to nitrate in the presence of molecular oxygen or nitrite reduction to NO under hypoxia^28, 29^. We have recently shown that preconditioning c-Kit positive hCPCs with the NO donor DETA-NO enhances cell survival through activation of survival signaling pathways^30^. The documented cytoprotective function of CYGB with regard to oxidative stress and NO would suggest that CYGB might play a role in regulating the therapeutic efficacy of human cardiac progenitor cells (hCPCs). Based on these premises, the aims of the present study were to investigate the potential role of CYGB in mediating the survival of hCPCs, and clarify the underlying mechanisms.

Using gain and loss of function approaches, we establish for the first time that the survival of hCPCs upon increased oxidative stress is significantly affected by CYGB protein levels. We also provide evidence that the upregulation of both anti-apoptotic and anti-oxidant molecules is associated with the cytoprotective effect of overexpressing CYGB. In addition, we identified that activated NFκB survival pathway, increased iNOS expression, and nitric oxide production are the potential molecular mechanisms associated with the cytoprotective effect of overexpressing CYGB in hCPCs. These findings suggest that CYGB plays an important role in protecting hCPCs against cell death induced by oxidative stress, providing a potential therapeutic target for enhancing the effectiveness of cardiac stem/progenitor cell therapy for ischemic heart disease.

## Results

### Overexpressing cytoglobin enhances hCPC survival and decreases apoptosis in response to oxidative stress

**Figure 1:**
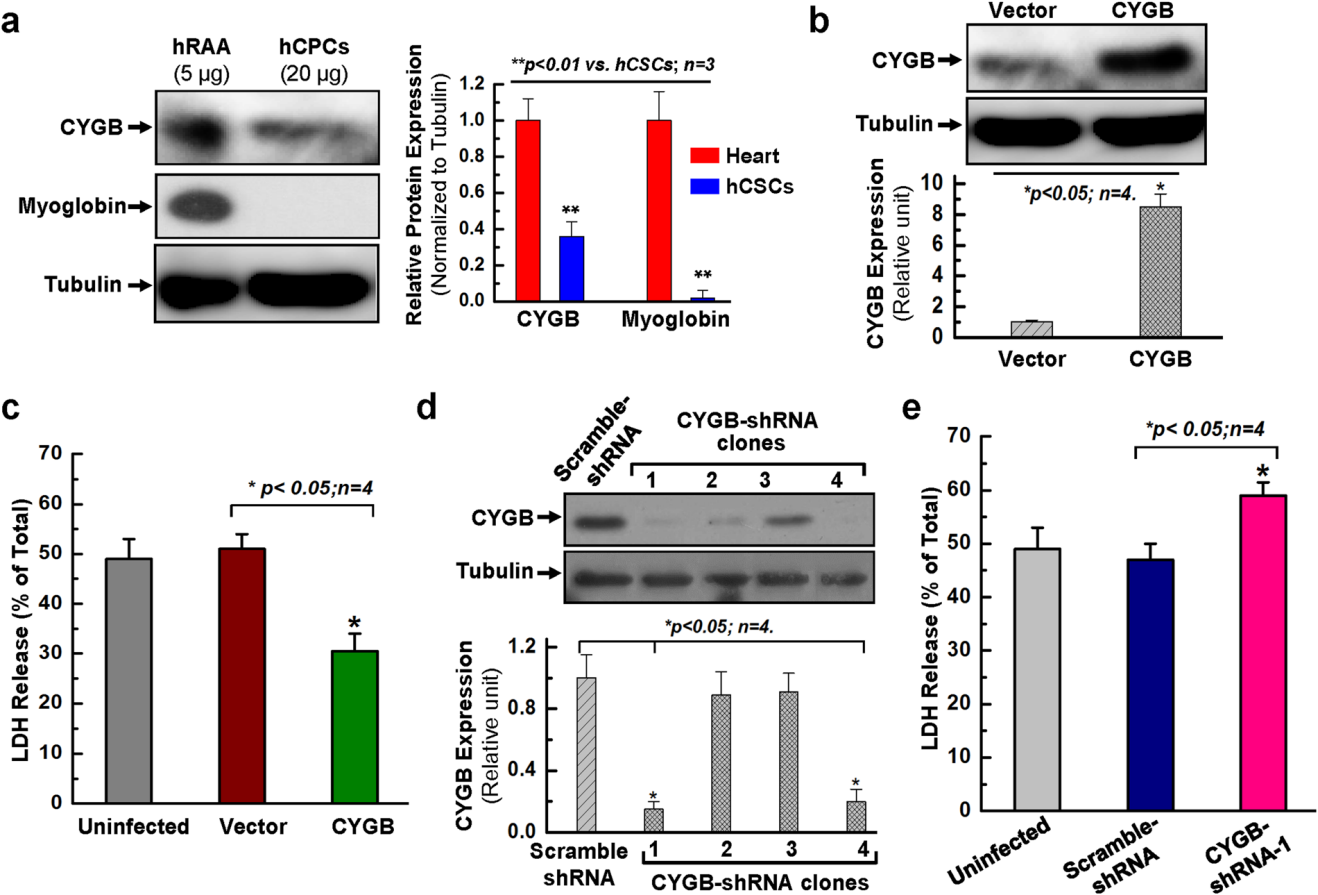
The expression of CYGB is associated with the cell survival ability against oxidative stress in hCPCs. (**a**) Representative images and quantitative data of Western blot showed that the expression of CYGB is observed in both hCPCs and human right atrial appendage (hRAA). However, the expression of myoglobin is not detectable in hCPCs compared to its abundant expression in hRAA. The total protein loading is 5 µg for hRAA and 20 µg for hCPCs. Full-length blots are presented in Supplemental Fig. S1. (**b**) Western blot confirmed that CYGB was overexpressed in hCPCs. Full-length blots are presented in Supplemental Fig. S1. (**c**) CPCs were infected with lentivirus expressing CYGB or vector for 24 h, challenged with 2 mM H_2_O_2_ for 3 h, and then evaluated by LDH assay. (**d**) Western blot confirmed that CYGB was knocked-down in hCPCs with shRNAs for clone #1&4. Full-length blots are presented in Supplemental Fig. S8. (**e**) Human CPCs were infected with lentivirus expressing shRNA against CYGB, or scramble shRNA for 24 h, challenged with 2 mM H_2_O_2_ for 3 h, then evaluated by LDH assay. Data presented in this figure were mean values on a ratio of H_2_O_2_-induced LDH release to total LDH in cells with standard error (means ± SEM). *Indicates *p* < 0.05 vs. control; **indicates *p* < 0.01 vs. control; n = 4 independent experiments.

Previous studies have indicated pro-survival functions for the non-canonical mammalian globin cytoglobin (CYGB) in response to oxidants and reactive oxygen species (ROS)^24–27^. In the present study, Western blot analysis revealed that CYGB was expressed in both hCPCs and human right atrial appendage (hRAA), although its expression level is significantly lower in hCPCs than that in hRAA. In contrast, myoglobin (MB) expression was evident in hRAA but absent in hCPCs (Fig. 1a). The expression of neuroglobin was not detectable in both hCPCs and hRAA (not shown). To examine whether CYGB plays a role in hCPC survival, we generated lentiviral particles to overexpress or knock down CYGB in hCPCs. Western blot analysis showed that lentiviral-mediated overexpression of CYGB resulted in an approximately 8-fold increase in CYGB protein levels (Fig. 1b). In contrast, over 80% knockdown was achieved in hCPCs infected with a lentivirus expressing shRNA against CYGB (Fig. 1d, clone# 1&4). The relative expression of CYGB in hRAA and multiple hCPC lines used in this study was also examined in one-gel Western blotting, which showed the similar expression level of CYGB in hCPCs infected with lentivirus particles expressing vector or scramble shRNA, and confirmed the significant overexpression or knocking down of CYGB in hCPCs (Fig. S1).

Next, hCPCs infected with the CYGB overexpression or knockdown lentiviral systems were incubated with 2 mM H_2_O_2_ for 3 hours and cell viability was evaluated using an LDH release assay. We found a 15% increase in cell viability upon overexpression of CYGB (Fig. 1c), and a statistically significant decrease in cell viability upon silencing of CYGB (Fig. 1e). Our laboratory recently showed that preconditioning c-Kit positive hCPCs with a nitric oxide (NO) donor, DETA-NO, enhances cell survival through activation of survival signaling pathways^30^. Interestingly, it was observed that the expression of CYGB was upregulated in a dose-dependent manner in response to DETA-NO preconditioning (Fig. S2a). Treatment with 250 µM DETA-NO also significantly increased cell survival as indicated by the decreased LDH release compared to no DETA-NO treatment (Fig. S2b). All these results suggested that the expression of CYGB is associated with increased cell survival in response to oxidative stress in hCPCs.

**Figure 2:**
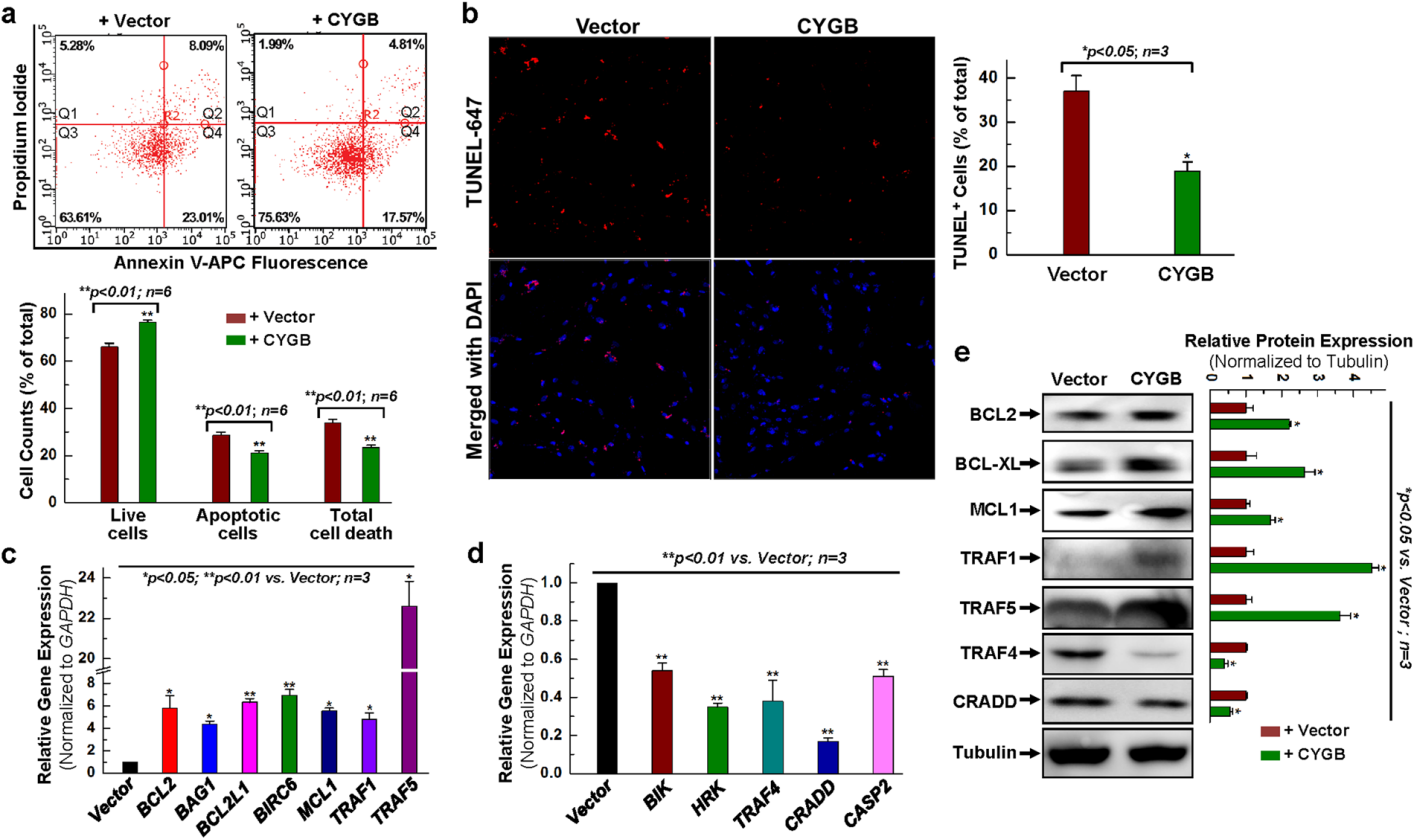
Overexpression of CYGB exhibits anti-apoptotic effect. (**a**) Human CPCs were infected with CYGB- or vector-lentivirus for 24 h, and challenged with 1 mM H_2_O_2_ for 1.5 h, then evaluated by Annexin/PI FACS assay with quantitative analysis. (**b**) Representative images for TUNEL staining and quantitative analysis for hCPCs infected with CYGB- or vector-lentivirus for 24 h, and challenged with 2 mM H_2_O_2_ for 2.5 h. (**c**) Examination of anti-apoptotic gene expression at the mRNA level after CYGB was overexpressed. (**d**) Examination of pro-apoptotic gene expression at the mRNA level after CYGB was overexpressed. (**e**) Representative images and quantitative data of Western blot showing anti-apoptotic and pro-apoptotic protein expression level after CYGB was overexpressed. Full-length blots are presented in Supplemental Fig. S10. *Indicates *p* < 0.05 vs. control; **indicates *p* < 0.01 vs. control; n = 3 independent experiments.

The effect of CYGB on hCPC apoptosis was next evaluated by Annexin V-APC and PI staining followed by flow cytometry. In these experiments, hCPCs were treated with 1 mM H_2_O_2_ for 90 min. Consistent with the results described for the LDH release assay, we found that overexpression of CYGB inhibited total cell death from 33.89 ± 1.54% in the control empty vector to 23.48 ± 0.91% in the cells overexpressing CYGB (Fig. 2a). Under these conditions, apoptosis was decreased by 25%. Other than the oxidative stress induced by H_2_O_2_, cell apoptotic assay was also performed for hCPCs subjected to hypoxia-reoxygenation induced injury (Fig. S3), confirming the enhanced cell survival ability after overexpressing CYGB in hCPCs. TUNEL assay was also performed in hCPCs subjected to oxidative stress by 1 mM H_2_O_2_ for 90 min. As shown in Fig. 2b, the number of TUNEL positive cells was significantly decreased in hCPCs with CYGB overexpression, confirming the cytoprotective role of CYGB. In addition, we found an increase in total cell death and apoptosis after CYGB was knocked-down (Fig. S4). Interestingly, overexpressing CYGB increased the cell survival ability in another subset of hCPC population, ALDH^br^-hCPCs (Fig. S5). This result indicated the cytoprotective effect of CYGB is not unique for c-kit^+^ hCPCs, which is also consistent with its cytoprotective effect in muscle progenitor cells^31^. Taken together, these results indicate that CYGB is expressed in hCPCs cells, and that changing its expression levels modulates hCPCs survival in response to H_2_O_2_-induced or hypoxia-reoxygenation induced oxidative stress, in a fashion that is consistent with a pro-survival and anti-apoptotic function for CYGB.

To start evaluating the mechanism by which CYGB may be regulating cell death and apoptosis in hCPCs, we performed a targeted array analysis of anti- and pro-apoptotic genes (Table S1) that might be changed upon overexpression of CYGB. Results from an apoptosis PCR primer library screening (Fig. S6a) indicated that there was a global increase in anti-apoptotic gene expression after CYGB overexpression compared with the empty vector group. Quantitative analysis of the results confirmed a significant (5~7-fold) increase in the expression of BCL2, BAG1, BCL2L1 (BCL-XL), BIRC6, MCL1 and TRAF1 in response to CYGB-overexpression, and a 23-fold increase for TRAF5 (Fig. 2c). The mRNA levels of pro-apoptotic genes, BIK, HRK, TRAF4, CRADD and CASP2 were all down-regulated in response to CYGB overexpression (Fig. 2d). This expression pattern of mRNA transcripts was confirmed at the protein level for anti-apoptotic (BCL2, BCL-XL, MCL1, TRAF1 and TRAF5) and pro-apoptotic genes (TRAF4 and CRADD) (Fig. 2e). In additional studies, we found no statistically significant differences in mRNA transcript levels for the cardiac lineage genes, including GATA4, NKX2.5, MEF2C and TBX5 after CYGB overexpression (Fig. S7), indicating no effect of CYGB on hCPCs potential for cardiac differentiation.

### Overexpressing cytoglobin results in decreased ROS generation

**Figure 3:**
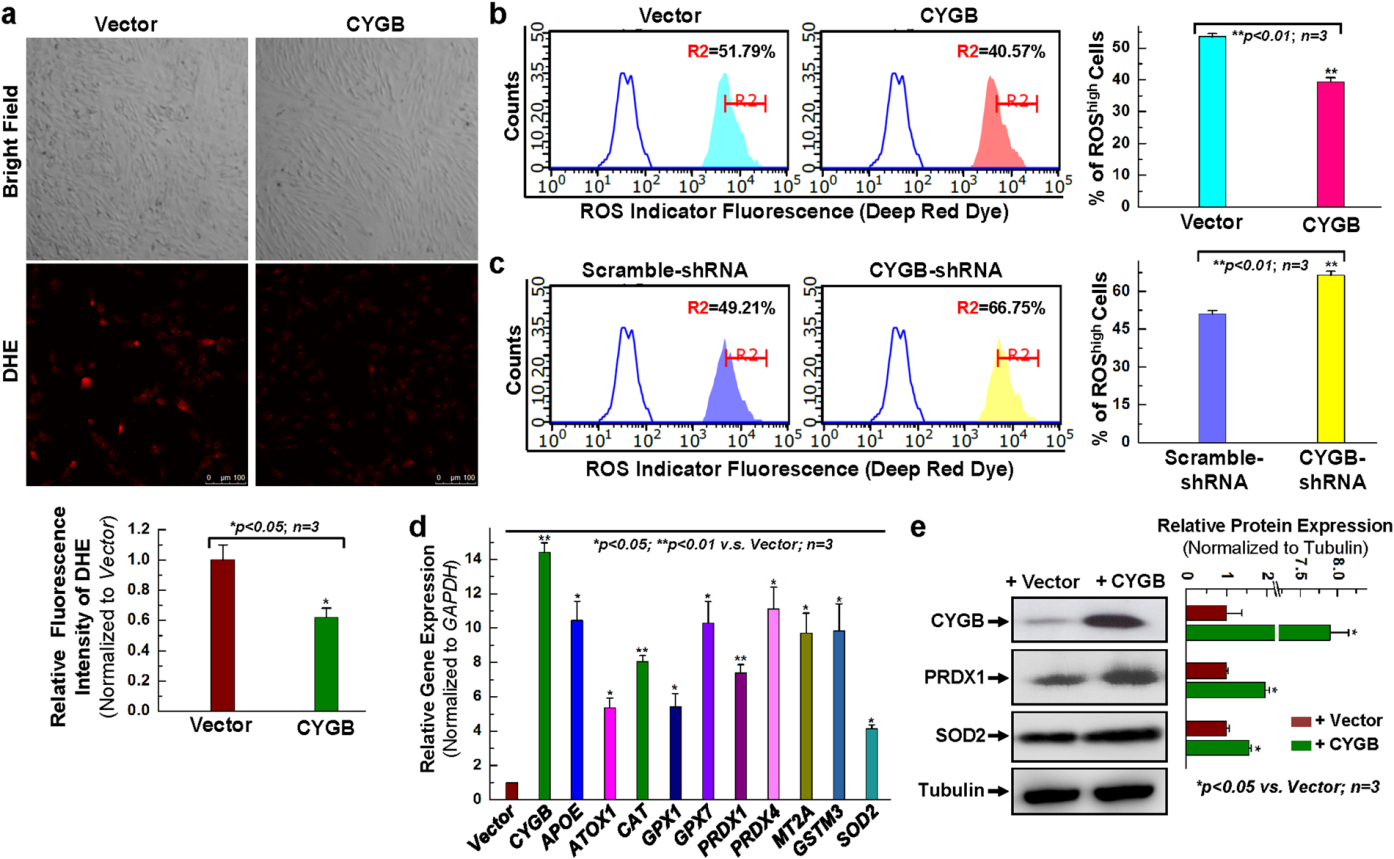
Overexpression of CYGB displays the anti-oxidant effect. (**a**) Representative images for DHE staining and quantitative analysis for hCPCs infected with CYGB- or vector-lentivirus for 48 hours. (**b**) Representative merged histogram and quantitative analysis for ROS measurement for hCPCs overexpressing CYGB vs. vector control by the FACS analysis with the ROS Detection Assay Kit (Deep Red Fluorescence). (**c**) Representative merged histogram and quantitative analysis for ROS measurement for hCPCs expressing shRNA against CYGB vs. scramble shRNA. The open blue traces are unstained cells as negative controls in panel b and c. (**d**) Examination of anti-oxidant gene expression at the mRNA level after CYGB was overexpressed. (**e**) Representative images and quantitative data of Western blot showing anti-oxidant protein expression level after CYGB was overexpressed. Full-length blots are presented in Supplemental Fig. 11. *Indicates *p* < 0.05 vs. control; **indicates *p* < 0.01 vs. control; n = 3 independent experiments.

In the next set of experiments, we evaluated whether altering CYGB levels could change the intracellular redox state of hCPCs as previously shown for other systems. First, the fluorescent imaging with DHE staining demonstrated that overexpressing CYGB significantly decreased intracellular ROS in hCPCs at resting state (Fig. 3a). Next, we examined the oxidation of the fluorescent indicator, Deep Red Dye (followed by the analysis of flow cytometry). An increase in the fluorescence intensity for Deep Red Dye suggests an increase in oxidation. We found that overexpression of CYGB decreased the oxidation of Deep Red Dye; while silencing CYGB increased the oxidation of Deep Red Dye suggesting that CYGB expression minimizes intracellular oxidation, possibly by reducing ROS bioavailability (Fig. 3b,c).

To expand on these results, we screened for the expression of genes classically associated with anti- and pro-oxidant functions using an oxidative stress targeted PCR primer library (Table S2). We found that there was a global increase in antioxidant pathways upon overexpression of CYGB (Fig. S6b) and establish a 5~15 fold increase in expression of APOE, ATOX1, CAT, GPX1, GPX7, PRDX1, PRDX4, MT2A, GSTM3 and SOD2 (Fig. 3d). We confirmed increased expression at the protein level for PRDX1 and SOD2 (Fig. 3e). All together, these results support past studies in other cell systems and indicate that CYGB may control intracellular redox biochemistry, in part through regulation of primary intracellular antioxidant systems.

### Up-regulation of NFκB signal pathway following overexpressing cytoglobin

**Figure 4:**
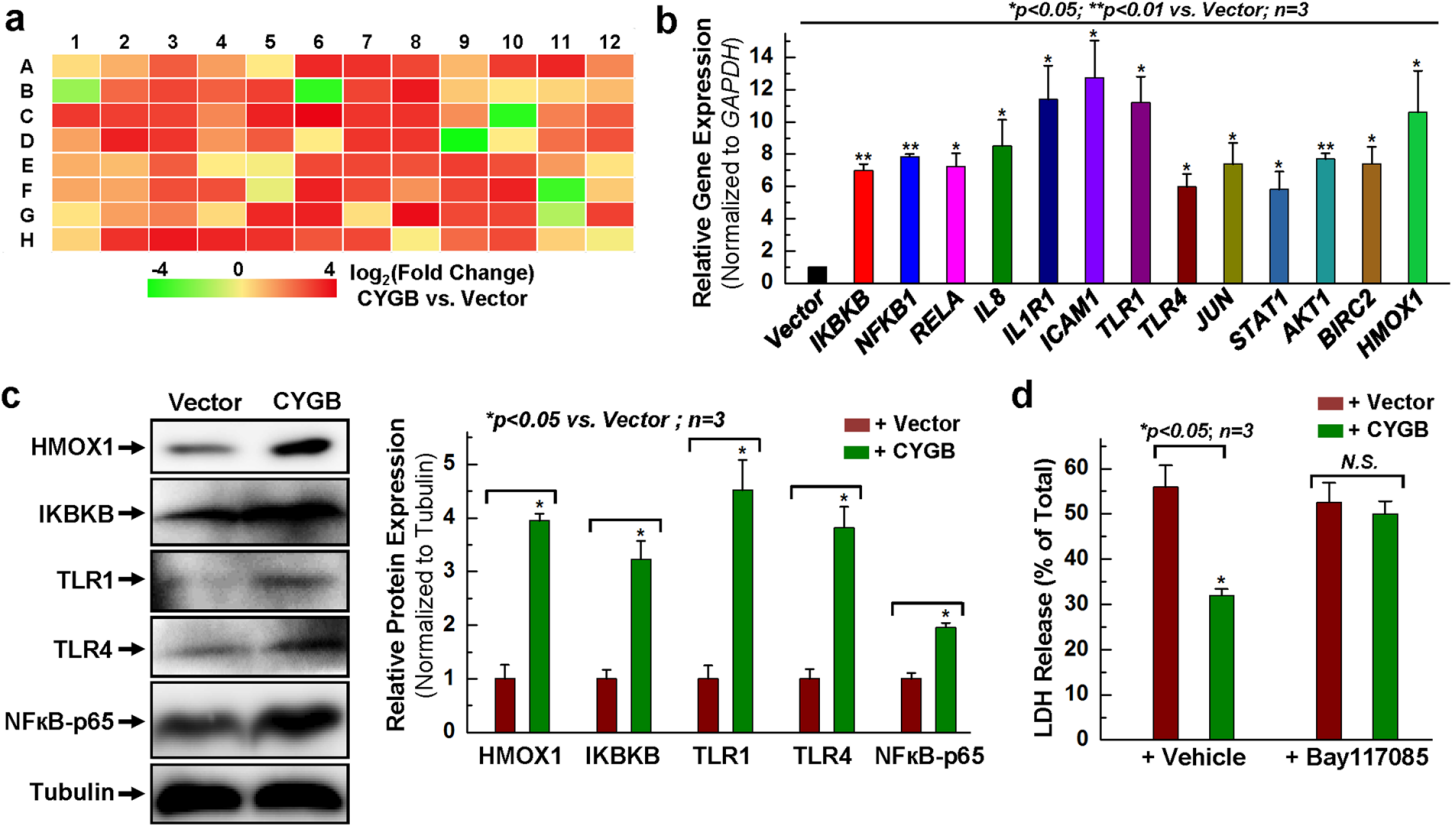
Up-regulation of NFκB signal pathway following overexpressing CYGB. (**a**) Heat map of NFκB library screening by real time PCR array in response to CYGB overexpression. List of genes in this library was shown in the Supplemental Table S3. (**b**) Examination of NFκB gene expression at the mRNA level after CYGB was overexpressed. (**c**) Representative images and quantitative data of Western blot showing the expression of NFκB-associated proteins after CYGB was overexpressed. Full-length blots are presented in Supplemental Fig. S12. (**d**) LDH release assay after Bay117085 (5 µM) was applied to inhibit NFκB activity compared with vehicle controls. *Indicates *p* < 0.05 vs. control; **indicates *p* < 0.01 vs. control; n = 3 independent experiments.

In addition to pro-inflammatory functions, the transcription factor NFκB is well-known for its role in promoting cell survival^30, 32, 33^. In the following experiments, we tested the specific hypothesis that NFкB is required for the cytoprotective effect associated with CYGB overexpression in hCPCs. To this end, evidence for NFκB-dependent gene expression was investigated by qPCR using an NFκB PCR primer library screen (Table S3). As shown in Fig. 4a, there was a global and massive increase in NFκB-related gene expression after CYGB overexpression, compared to the control vector group. Quantitative PCR analysis confirmed a 6~13-fold increase in the gene expression of IKBKB, NFΚB1, RELA, IL8, IL1R1, ICAM1, TLR1, TLR4, JUN, STAT1, AKT1, BIRC2 and HMOX1 (Fig. 4b). The protein levels of HMOX1, IKBKB, TLR1, TLR4 and NFκB-p65 were significantly upregulated upon overexpressing CYGB in hCPCs (Fig. 4c).

**Figure 5:**
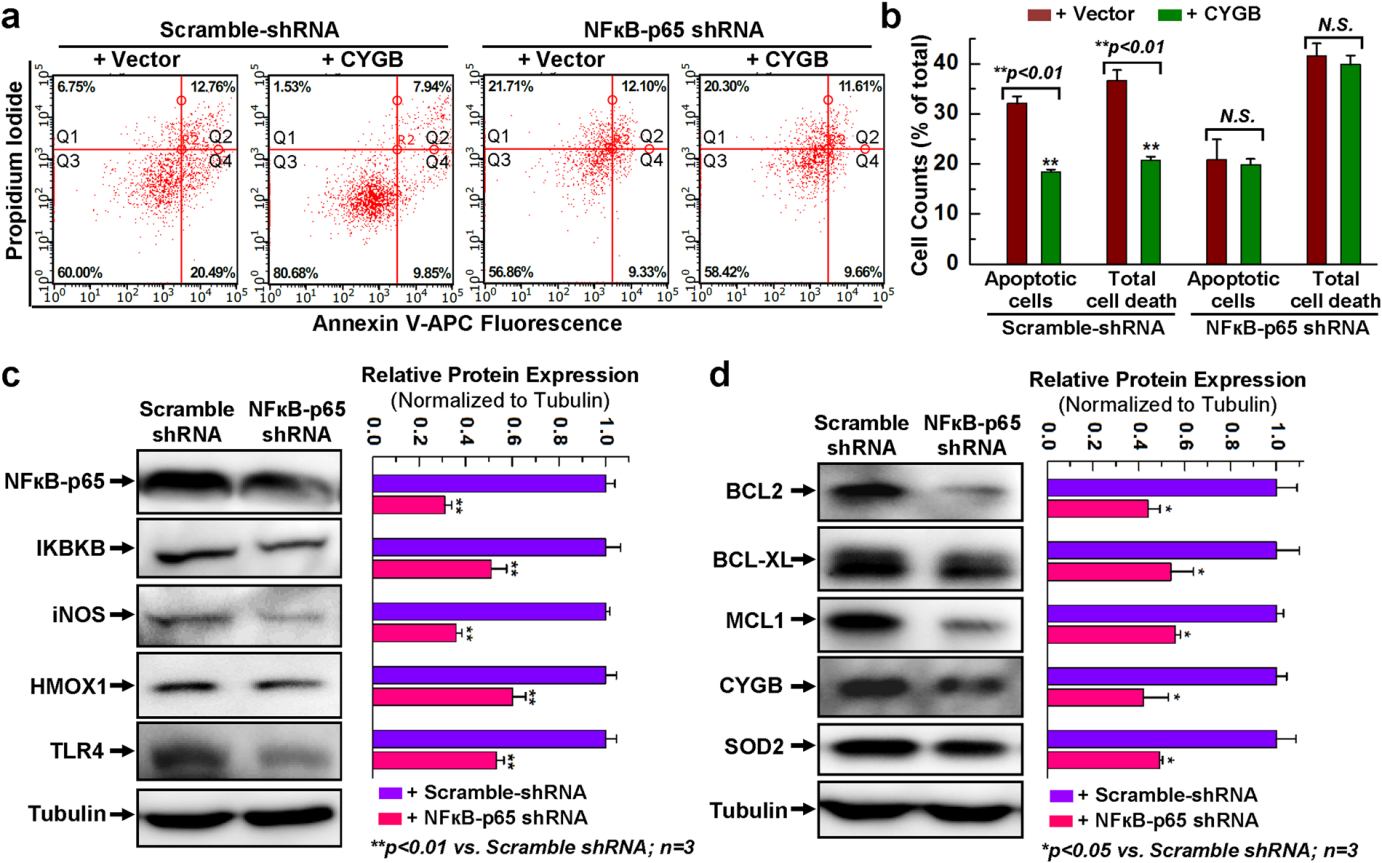
Disruption of NFκB expression abolished the cytoprotective effect of overexpressing CYGB. (**a**) Representative FACS analysis with annexin V/PI staining showing H_2_O_2_-induced apoptosis in hCPCs stably expressing scrambled or NFκB-p65 shRNA following with or without CYGB overexpression. (**b**) Quantitative data analysis for panel a. (**c**) Representative images and quantitative data of Western blot showing NFκB-related protein expression levels after NFκB was knocked-down. Full-length blots are presented in Supplemental Fig. S13. (**d**) Representative images and quantitative data of Western blot showing anti-apoptotic and anti-oxidant protein expression levels after NFκB was knocked down. Full-length blots are presented in Supplemental Fig. S14. *Indicates *p* < 0.05 vs. control; **indicates *p* < 0.01 vs. control; n = 3 independent experiments.

Next, cytotoxicity was evaluated following pharmacological inhibition of the NFκB pathway. Treatment of the cells with H_2_O_2_ resulted in LDH release that amounted to ~56% of maximum release. This was almost halved upon overexpression of CYGB in hCPCs (Fig. 4d). Most significantly, pretreatment of the cells with the NFκB inhibitor Bay117085 before application of H_2_O_2_ reversed the cytoprotective effect of CYGB overexpression, suggesting a role for NFкB in regulating CYGB-mediated cytoprotection (Fig. 4d). To confirm these results we used a previously established hCPC line with stable knockdown of NFκB (denoted NFkB-p65 shRNA in Fig. 5) and compared it to its control (Scramble-shRNA) using Annexin V/PI staining and flow cytometry analysis^30^. Following H_2_O_2_ treatment of the controls (Scramble-shRNA), approximately 33% of the cells were apoptotic (Annexin V positive) and 19% were dead (PI positive). While there was diminished apoptosis (20%) with the NFkB-p65 shRNA cells, there is no significant difference for both the apoptotic cells and the total cell death between the vector and CYGB group with NFkB-p65 knocking down, indicating the cytoprotective effect associated with CYGB overexpression was lost when the number of Annexin-V or PI positive cells was considered (Fig. 5a,b).

Next, we established through Western blot analysis that the expression of genes requiring NFкB was indeed inhibited in the hCPC cell line stably expressing the NFkB-p65 shRNA (Fig. 5c). These genes included IKBKB, HO-1, TLR4, and iNOS. Most significantly, the expression of anti-apoptotic BCL-2 family members, which we had found to be increased upon CYGB overexpression, were downregulated upon molecular inhibition of NFкB, concomitant with CYGB and SOD2 (Fig. 5d). Overall, our results indicate that pharmacological and molecular manipulations leading to the inactivation of NFкB diminished CYGB-mediated cytoprotection and that this was potentially mediated through the regulation of anti-apoptotic factors such as BCL-2 family members.

### Reciprocal role for iNOS-derived nitric oxide and CYGB in regulating hCPCs survival

**Figure 6:**
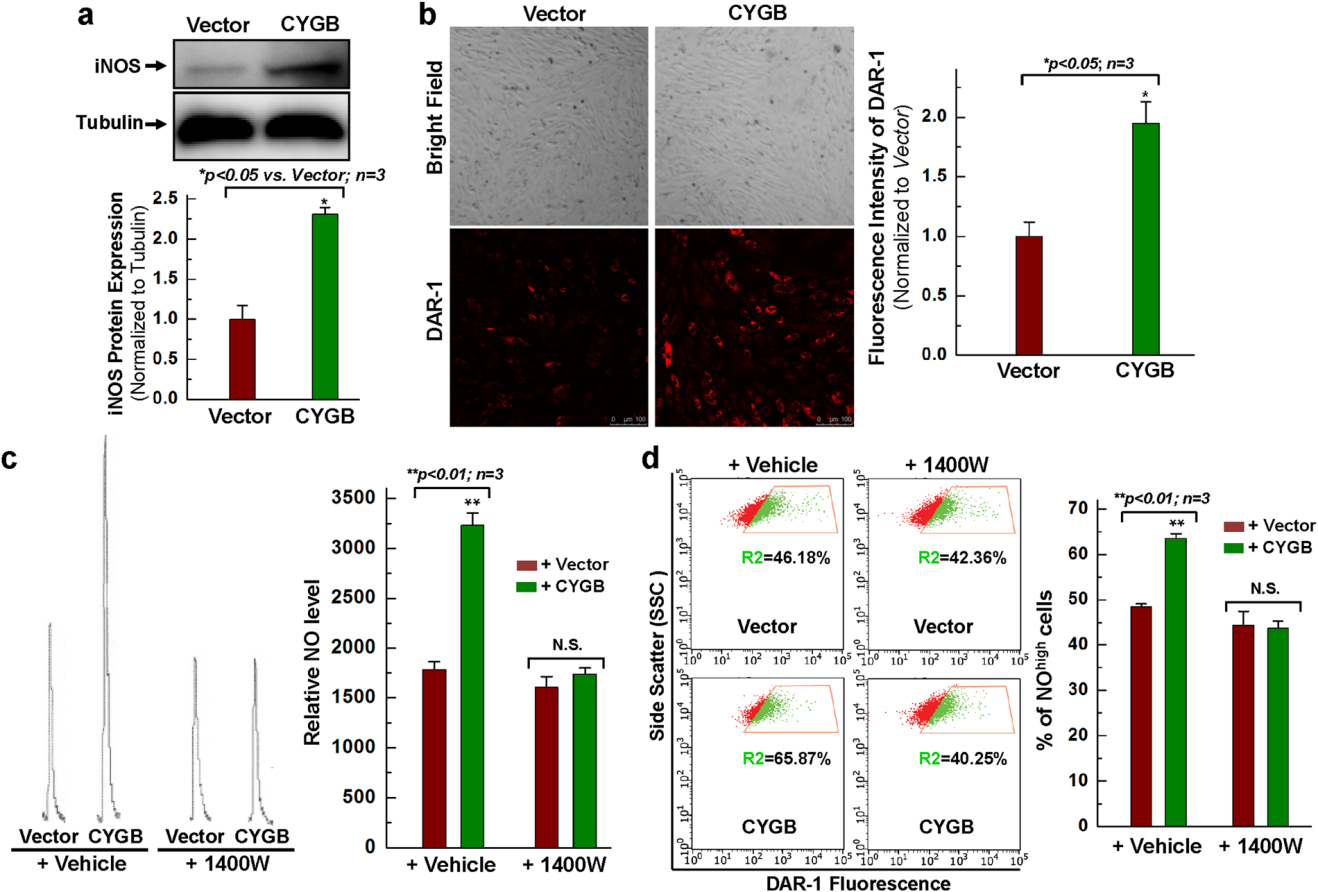
Overexpressing CYGB increases the iNOS expression and nitric oxide production, which effect is abolished by iNOS inhibitor 1400 W. (**a**) Representative images and quantitative data of Western blot showing iNOS expression in hCPCs overexpressing CYGB vs. vector control. Full-length blots are presented in Supplemental Fig. S15. (**b**) Representative DAR-1 staining images and quantitative analysis for hCPCs infected with CYGB- or vector-lentivirus for 48 hours. (**c**) Representative images and quantitative analysis showing the traces for the measurement of nitric oxide levels by chemiluminescence in hCPCs overexpressing CYGB vs. vector control following the treatment with 100 µM 1400 W or vehicle. See experimental details in the Material and Methods Section. (**d**) Representative FACS analysis with DAR-1 staining showing the NO level in hCPCs overexpressing CYGB vs. vector control following the treatment with 100 µM 1400 W or vehicle. *Indicates *p* < 0.05 vs. control; **indicates *p* < 0.01 vs. control; n = 3 independent experiments.

We already found that hCPCs express iNOS (Fig. 5c). However, the expression of eNOS and nNOS is not detectable in hCPCs (Fig. S9). Because previous studies have indicated that CYGB may be involved in nitric oxide (NO) metabolism^28, 29^, we wanted to determine whether CYGB could reciprocally regulate iNOS expression and activity. We found that overexpression of CYGB in hCPCs cells increased iNOS protein expression by approximately 2.5 fold over basal levels (Fig. 6a). This coincided with an increase in NO production as assessed by fluorescent imaging with a nitrogen oxide sensor, DAR-1 (Fig. 6b) and the chemiluminescence analysis of NO metabolites (Fig. 6c). Further evaluation with FACS analysis for the live cells stained with DAR-1 confirmed the elevated NO production in hCPCs with CYGB overexpression (Fig. 6d). In either case, the effect of CYGB on indicators of NO production could be reversed through pharmacological inhibition with the iNOS specific inhibitor 1400 W (Fig. 6c,d).

**Figure 7:**
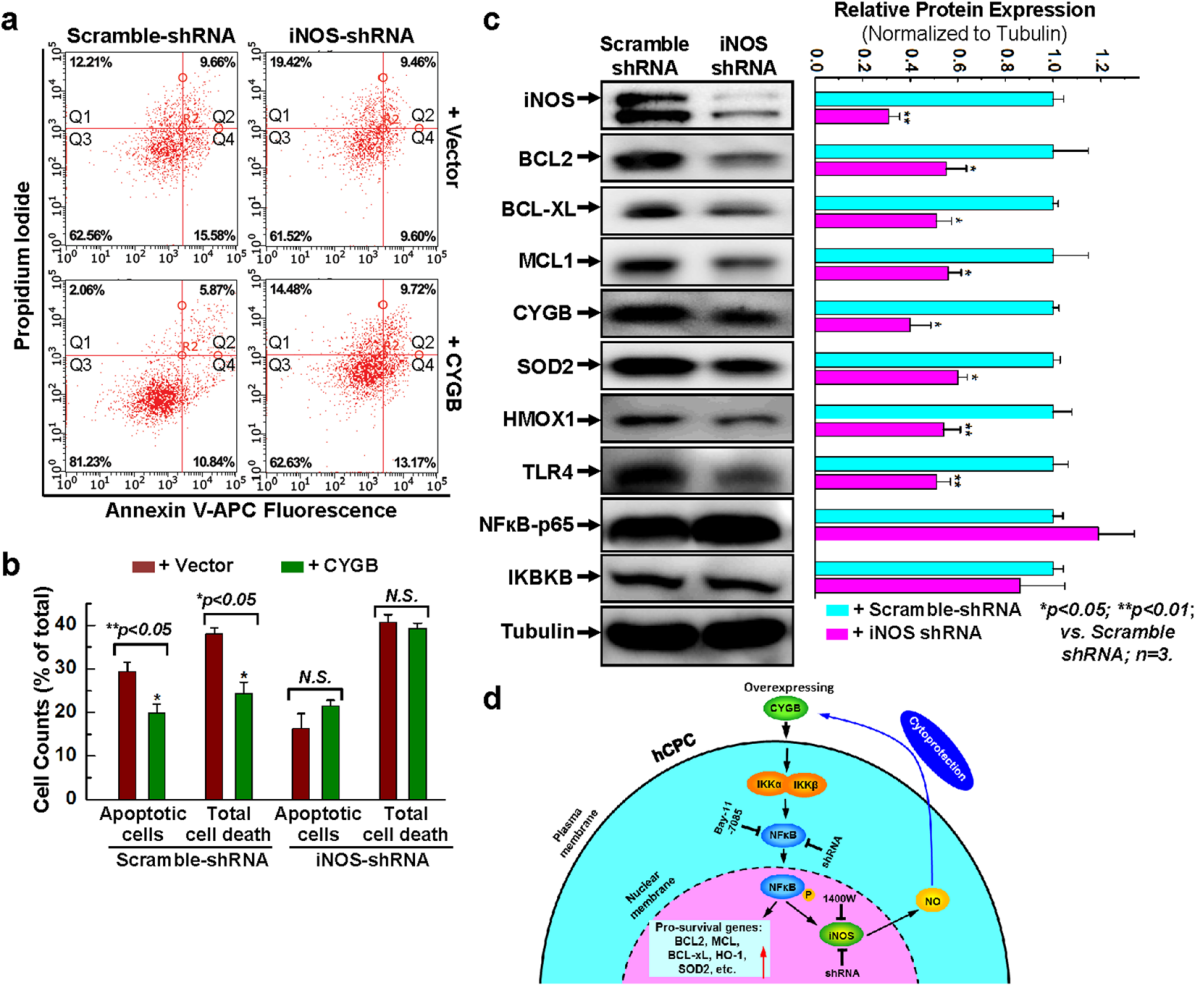
Knocking down iNOS diminishes the cytoprotective effect of overexpressing CYGB. (**a**) Representative FACS analysis with annexin V/PI staining showing H_2_O_2_-induced apoptosis in hCPCs stably expressing scrambled or iNOS shRNA following with or without CYGB overexpression. (**b**) Quantitative data analysis for panel a. (**c**) Representative images and quantitative data of Western blot showing the expression of anti-apoptotic and anti-oxidant proteins after iNOS was knocked down. Full-length blots are presented in Supplemental Fig. S16. (**d**) Descriptive diagram of proposed molecular mechanism for CYGB regulated hCPC survival. *Indicates *p* < 0.05 vs. control; **indicates *p* < 0.01 vs. control; n = 3 independent experiments.

To determine whether iNOS, downstream of NFкB, played a role in the cytoprotective effect associated with CYGB, we overexpressed CYGB in an hCPC cell-line with stable knockdown of iNOS (iNOS-shRNA). As already shown, treatment of a control cell line (Scramble-shRNA; Fig. 7a,b) with H_2_O_2_ resulted in significant amounts of apoptotic and dead cells as assessed by Annexin-V/PI staining and flow cytometry, an effect partially inhibited upon overexpression of CYGB. However, the cytoprotective effect of CYGB overexpression was lost in the iNOS-shRNA cells. Decreased iNOS protein expression in the iNOS shRNA cell line was confirmed by WB blotting (Fig. 7c). We found that these cells expressed lower levels of CYGB but also had decreased levels of anti-apoptotic BCL2 family members (BCL-2, BCL-XL, and MCL1) and decreased levels in protein expression downstream of NFκB including HO-1 and TLR4. This occurred in the absence of any change in the p65 subunit of NFκB or IKBKB (Fig. 7c).

## Discussion

The main and novel result presented in this study is that overexpressing CYGB in hCPCs leads to a significant increase in cell survival in response to H_2_O_2_ challenge. Accordingly, silencing of CYGB increases cell death upon treatment of hCPCs cells with H_2_O_2_. We have also established that the cytoprotective effect of CYGB is most likely not direct (i.e. direct reaction with H_2_O_2_ by CYGB) but rather involves the regulation of gene expression downstream of NFκB. This culminates in a feedforward loop to promote CYGB expression through increased levels of iNOS and increased production of NO. Previous studies have shown expression of CYGB in the cardiovascular system, including in arteries in vascular smooth muscle cells^28^ and in the heart^34^, although in the latter clear demonstration of CYGB expression in cardiomyocytes was lacking. In the present study, we show that human-derived cardiac progenitor cells (hCPCs) express CYGB but lack myoglobin (MB), the primary globin expressed in adult cardiomyocytes. The present study is the first to undertake a comprehensive analysis of CYGB function in cardiac progenitor cells.

Cytoglobin is a hexacoordinated hemoglobin that reversibly binds diatomic gases including molecular oxygen, carbon monoxide, and nitric oxide (NO). Based on initial structural and functional characterization of CYGB and association of CYGB expression *in vivo* with pro-fibrotic conditions that may be conducive to an increase in oxidant load, it was proposed that CYGB may confer a survival advantage through direct scavenging of reactive oxygen species (ROS) such as H_2_O_2_. In the present study, we find that changing CYGB levels in hCPCs alters indicators of intracellular redox states in a fashion that is consistent with an antioxidant function for CYGB. However, we believe that this effect is indirect. Past biochemical studies have failed to identify a specific antioxidant activity associated with CYGB and peroxidase activities previously characterized are not different from other hemoglobins. Most importantly, we found that overexpression of CYGB results in the upregulation of a number of key antioxidant proteins, including PRDX1, SOD2, and HO-1. The role of peroxiredoxins including PRDX1 as primary intracellular peroxidases has been clearly demonstrated in various systems and the concerted increase of PRDX1, SOD2, and HO-1 might be sufficient to explain the antioxidant effect of CYGB. Changes in expression levels of key antioxidant enzymes have also been documented in other studies in response to changes in CYGB levels^31, 35^.

Apoptosis is one of many key contributors to the loss of cardiac stem/progenitor cells induced by ischemia/reperfusion or other insults. Up-regulation of anti-apoptotic gene/protein and down-regulation of pro-apoptotic gene/protein have been reported to provide cytoprotection during myocardial repair^30, 36^. Recent studies have also indicated a role for CYGB in regulating apoptosis^31, 37, 38^ in part through increased expression of anti-apoptotic factors, although key regulators such as anti-apoptotic BCL-2 family members had not been documented. In the present study, we show that CYGB expression in hCPCs confers anti-apoptotic functions and this is associated with the increased expression of core anti-apoptotic factors such as BCL-2, BCL-XL, and MCL1. Pro-apoptotic proteins such as TRAF-4 and CRADD were also downregulated. Our results strongly suggest that CYGB plays a cytoprotective role against cell death induced through oxidative stress, at least in part by promoting an anti-apoptotic phenotype.

In addition to binding molecular oxygen and NO, CYGB supports the deoxygenation of NO to nitrate^28, 39^. There are also some indications that CYGB may regulate NO dependent cytostasis and cytotoxicity^28, 35^. In contrast, some studies indicate that iNOS-dependent NO generation contributes to cell survival in osteoclasts^40^, cancer cells^41^, peripheral blood cells^42^, and bone marrow-derived mesenchymal stem cells^43^. In light of the potential reciprocal interactions between NO and CYGB, we decided to explore if CYGB and iNOS could be co-expressed in hCPCs and whether this co-expression had some functional significance. Surprisingly, we found that CYGB increased iNOS expression at both the mRNA and protein levels. This coincided with an increase in NO production, an effect that could be reversed upon pharmacological inhibition of iNOS. Most importantly, the cytoprotective effect associated with CYGB expression was abolished upon silencing of iNOS. Our results reinforce the potential link between CYGB, iNOS, and NO. In this context, the expression of iNOS is strongly regulated by the transcription factor NFκB^44, 45^. Our results indicate that overexpressing CYGB in hCPCs increased the expression of NFκB-related genes including HMOX1, IKBKB, TLR1, TLR4 and NFκB-p65. In addition, pharmacological and molecular inhibition of NFкB activity diminished the cytoprotective effect induced by overexpressing CYGB. Overall our study suggests a feedforward mechanism whereby CYGB may promote survival by increasing NFκB-dependent iNOS expression, to increase NO production, which in turn maintains CYGB expression (Fig. 7d). How exactly CYGB regulates NFкB is unclear at present time and will require additional studies.

In the past decade, the potential of c-kit^+^/Lin^−^ CPCs for myocardial repair has been tested *in vivo* using different models of myocardial damage in various mammalian animals^2, 3, 46, 47^. An early-stage human clinical trial has also shown encouraging results in the use of autologous CPCs in patients with heart failure^48^. With emerging evidence, c-kit^+^ CPCs are thought to be necessary and sufficient for cardiac regeneration and repair^49^. However, the controversial debate about CPC commitment to cardiomyocytes has been recently raised. The results from lineage-tracing studies suggest that the proportion of cardiomyocytes derived from c-kit^+^ cells are limited^50, 51^. Though those interesting findings urge us to redefine the physiological role of c-kit^+^ CPCs in the heart, it is no doubt that there is the beneficial effect of using c-kit^+^ donor cells to improve cardiac function, which therapeutic effect of cardiac progenitor cell therapy may mainly due to the mechanism of paracrine effect, since global increase of cytokine expression was observed in preconditioned cells^36^. The focus our current research is to enhance the effectiveness of cardiac progenitor cell therapy by improving cell survival via increasing the expression of cytoglobin. Our rationale is that more cell survival will result in more cytokine release, which may lead to the promotion of endogenous cardiac stem/progenitor cell survival or regeneration. However, elucidating the potential mechanisms for the therapeutic effect of cardiac progenitor cell therapy will require a considerable effort, which is beyond the scope of the present study.

In summary, this is the first conclusive study to address the pivotal role of CYGB in suppressing oxidative stress-induced cell apoptosis in hCPCs. Our studies would also suggest for the first time that CYGB function may involve a regulatory loop associated with NFκB/iNOS signaling pathways that regulate downstream anti-apoptotic gene expression. Our functional studies demonstrate that CYGB expression in hCPCs enhances cell survival. We propose that this strategy may be used as a simple and effective strategy to improve the efficacy of CPC-based therapies for heart disease. Future studies will require to examine the *in vivo* survival capacity of hCPCs upon upregulation of CYGB, and conduct a thorough analysis of the molecular mechanisms underlying the potential cytoprotective function of CYGB following acute myocardial infarction.

## Material and Methods

### Reagents

Collagenase II was from Worthington Biochemical. Ham’s F12 medium was from Invitrogen. Fetal bovine serum (FBS) was obtained from Hyclone. Antibody to CYGB was from Miami Valley Biotech; antibodies to PRDX1, TRAF1, TRAF4, CRADD, IKBKB, TLR1 and TLR4 were from R&D Systems; antibodies to SOD2 and HMOX-1 were from Santa Cruz; antibodies to BCL2, BCL-XL, MCL-1, NFkB-p65 were from Cell Signaling; antibody to TRAF5 was from Enzo Life Sciences, Inc. Please see the Table S4 for the detailed information for the antibodies used in the immunoblot analysis. Quantitative PCR primers for target genes were obtained from Real Time Primers, LLC. Unless indicated otherwise, chemicals used in experiments were purchased from Sigma.

### Harvesting of human c-kit^+^ CPCs

Human cardiac progenitor cells (hCPCs), expressing c-kit cell surface marker, were isolated and purified using atrial appendages from patients during open-heart surgery at Albany Medical Center. The procedures for isolating hCPCs were exactly followed as described previously^30, 52, 53^. Briefly, right atrial tissues (100 to 400 mg) were minced and enzymatically digested with collagenase II (30 U/ml) at 37 °C in a shaking water bath. During the incubation, chopped tissues were mechanically disturbed by gently pipetting several times. After 1 h of incubation, undigested clumps were separated by gravity on ice for 10 min, and the supernatant was carefully transferred into a 15-ml tube. Dissociated cells from digestion was collected by centrifuge at 1200 rpm for 5 min. Cell pellet was suspended and cultured in Ham’s F12 medium supplemented with 10% FBS, 10 ng/ml human basic FGF, 0.005 U/ml human erythropoietin, 0.2 mM L-glutathione, 100 U/ml penicillin and 100 μg/ml streptomycin. Cells were maintained in a humidified environment at 37 °C and 5% CO_2_. The day after, the CPC growth medium was refreshed and adherent cells were cultured with medium change every other day. On reaching around 80% of confluence, cells were sorted using the c-kit MACS kit according to manufacturer’s instructions (Miltenyi Biotec), expanded, and characterized by FACS analysis to obtain lineage-negative hCPCs. Cell characterization has been performed as described previously^30, 36, 52, 53^. These cells were frozen and stocked around passage 6–7 in order to have enough cells for all the proposed studies. Human CPC lines from the different donors were used at passage 8–15 to perform all the *in vitro* experiments in this study. And these cells could maintain the stemness with over 80% of c-kit positive up to 20 passages as described previously^54^. ALDH^br^-hCPCs were sorted as described previously^55^.

### Lentiviral infection of hCPCs

ORF expression clone for CYGB (purified plasmid), empty control vector for pReceiver-Lv202, shRNA scrambled control clone for psi-LVRH1GP, and shRNA clone set against human CYGB were purchased from GeneCopoeia. Lentiviruses were packaged by Lenti-Pac FIV expression packaging kit according to the manufacturer’s instructions (GeneCopoeia). Split hCPCs to be infected at proper density in a 6-well tissue culture plate in 2 ml of F-12 complete media. Seed the proper number of plates for the number of experiments being run. The proper controls and replicates were included. Cells were incubated in a 37 °C incubator at 5% CO_2_ overnight undisturbed. An infectious viral titer needs to be determined prior to infection in order to calculate the amount of viruses to be added. Polybrene were added to the 1 mL of virus/media for a final concentration of 5 ug/mL. Finally, 1 mL of media containing virus was added to the plated cells.

### Specific knockdown of NFκB and iNOS in hCPCs

To specifically knock down NFκB or iNOS in hCPCs, we established the stable cell lines expressing shRNA against NFκB or iNOS in hCPCs using shRNA lentiviral particles according to the manufacturer’s instructions (Santa Cruz Biotechnology). Scrambled shRNA lentiviral particles were also used to infect hCPCs as negative control. Human CPCs were seeded at the density of 1 × 10^5^ per well in a 6-well plate 24 h prior to infection. At 50–60% confluence, cells were refreshed with 2 ml of the culture medium followed by the direct addition of 5 μg/ml polybrene and 20 μl of lentiviral particles with gentle orbital shaking. The day after, medium was replaced with normal growth medium for recovery. 48 h after infection, 5 μg/ml puromycin was applied to the culture medium in both infected and non-infected hCPCs until all non-infected cells died eventually (2–3 days). In order to obtain stable transfected cells, hCPCs were further cultured in puromycin-containing growth medium for up to 10 days. The efficiency of gene knockdown was subsequently confirmed by Western blot.

### Cell viability assay

LDH release assay, a simple approach to measure cellular membrane integrity, was applied to determine the oxidative stress-induced cell death in hCPCs. The procedures were exactly followed according to manufacturer’s instructions from Cytotoxicity LDH Detection kit (Takara). The day before CYGB-lentivirus infection, hCPCs were seeded at the density of 1 × 10^4^ per well in a 96-well plate. After 24 h infection, cells were exposed to 2 mM H_2_O_2_ for 3 hours, which condition was optimized based on previous studies^30^. The plate was then centrifuged at 250 × g for 10 min. 100 μl of the supernatant was collected and mixed with the equal volume of pre-prepared solution (catalyst/dye buffer = 1:45) for 30 min at room temperature in a 96-well plate. The absorbance of samples at 490 nm was measured using a Bio-Rad iMarkTM microplate reader. The percentage of LDH release for each sample was exhibited by comparing to the absorbance value from cells pretreated with 0.5% Triton X-100.

### TUNEL assay

Cell apoptosis was identified by the terminal deoxtnucleotidyl transferase (TdT) mediated dUTP nick-end labeling (TUNEL) method, using the TUNEL Andy FluorTM 647 Apoptosis Detection kit (Genecopoeia). Procedures were performed according to manufacture instructions. Briefly, hCPCs were plated in 24-well plates with 0.2% gelatin coated 12 mm round cover slip glass. After 36 hours of infection with lentivirus expressing CYGB or vector, hCPCs were treated with 2 mM H_2_O_2_ in serum-free F12 medium for 2.5 hours. Cells were washed with PBS twice and fixed with 4% formaldehyde for 30 mins at 4 °C. A positive control and a negative control were used by following the manufacture protocol. All samples were incubated with TdT reaction cocktail for 60 mins at 37 °C in dark. Then washed 3 times with PBS solution containing 3% BSA, then incubated with FluorTM 647-Streptavidin staining solution for 30 mins at room temperature in dark. 12 fluorescence images for each group were taken with a Leica fluorescence microscope.

### FACS analysis for apoptotic assay

Cell apoptosis was investigated by dual staining of hCPCs with APC-conjugated Annexin V and Propidium Iodide (PI), using Annexin V/Dead cell apoptosis kit (Invitrogen). Briefly, hCPCs were plated at a density of 2 × 10^5^ per well in a 6-well plate prior to CYGB-lentivirus infection. After 24 h of infection, hCPCs were challenged with or without 1 mM H_2_O_2_ in serum-free F12 medium for 90 min, which is a relatively moderate condition compared to the LDH release assay in order to induce early and late cell apoptosis. Cells were then detached by TrypLE solution (Invitrogen), washed twice with PBS, and suspended in 100 μl of 1x annexin-binding buffer supplemented with 1 μg/ml PI and 5 μl of Annexin V, Alexa Fluor 647 conjugate solution (Invitrogen). After 15 min’s incubation at room temperature, 400 μl of 1 annexin-binding buffer was added with gentle mixing, and samples were immediately analyzed by the Guava EasyCyte™ System (EMD Millipore Corporation, Inc). Data was analyzed by GuavaSoft™ Module software (EMD Millipore Corporation, Inc). The four quadrants (Q1, Q2, Q3, and Q4) are marked in all FASC analysis. Cells positive for Annexin V only (Q4), or double positive for both Annexin V and PI (Q2) were defined as apoptotic cells (Q2 + Q4); Cells positive for PI only (Q1) were defined as necrotic cells; and cells negative for both Annexin V and PI (Q3) were defined as live cells.

### Cell proliferation assay with BrdU incorporation

Human CPCs incorporated with BrdU were analyzed by flow cytometry to determine the effect of CYGB on cell proliferation ability as described previously^30, 36^. Briefly, hCPCs were infected with CYGB lentivirus for 24 h and then refreshed with growth medium supplemented with 10 μM BrdU. 12 h later, cells were detached by trypsinization, washed with PBS twice, and fixed with 70% ice-cold ethanol for 1 h on ice. Cells were then treated with 2 M HCl for 30 min followed by permeabilization with 0.5% Triton X-100 for 20 min. After sufficient 4x PBS washes, cells were blocked for 30 min, followed by staining with BrdU antibody in 1% BSA/PBS for 2 h, which was followed by the application of a secondary mouse-APC antibody for 1 h. Cells were subsequently diluted to an appropriate volume in PBS for flow cytometry analysis.

### ROS measurement

To evaluate ROS production by hCPCs, dihydroethidium (DHE) staining was employed according to manufacturer’s instruction as previously reported^56, 57^. Briefly, cells were loaded with DHE (1 mM, Invitrogen) for 10–20 min in dark. After washing twice with PBS, 12 fluorescence images for each group were taken by a fluorescence microscope (Leica). The intensity of DHE staining was quantified using the Image J software. Each experiment was repeated at least three independent times. A Cellular ROS Detection Assay Kit (Abcam) was also used to measure the intracellular ROS Level. Procedures were performed according to manufacture instructions. Human CPCs were detached by TrypLE solution and stimulated with H_2_O_2_ (1 mM) for 90 min. The cell-permeable ROS Deep Red Dye was then added to suspended cells with appropriate dilution (1:1000), and cells were maintained at 37 °C for another hour. After two PBS washes, the intensity of red fluorescence was determined by flow cytometry analysis.

### RNA isolation and quantitative real time PCR

Real-Time PCR was performed to determine the change of anti-apoptotic or NFκB pathway-related genes in response to overexpressing CYGB in hCPCs. Primers used in this study were obtained from RealTimePrimers, LLC. The total RNA of each sample was extracted and purified using AurumTM Total RNA Mini Kit (Bio-Rad). The quality and quantity of RNA were detected by a NanoDrop 2000C spectrophotometer (Thermo Scientific). The reverse transcription of 1 μg of RNA to cDNA was established using Bio-Rad iScriptTM cDNA synthesis Kit. Samples for Real-time PCR were prepared according to the manufacturer’ instructions of the iQ SYBR Green Supermix kit (Bio-Rad). Real-time PCR was run with a Bio-Rad iQ5 optical module. Cycling conditions were: 95 °C for 2 min as initial denaturation, 40 cycles of denaturation at 95 °C for 15 sec, and annealing/extension at 60 °C for 40 sec. Melt Curve analysis was set between 65 °C and 95 °C with 0.5 °C increments at 5 sec per step. In those experiments, GAPDH was used as internal control gene for quantitative RT-PCR expression analysis. Gene lists of the Pathway PCR Primer Library screening can be found on the website of RealTimePrimers for Human Apoptosis/Oxidative Stress/NFкB Signaling pathways.

### Immunoblotting

Western blot analysis was carried out according to protocol as described previously^30, 36^. After two times the ice-cold PBS washes, cells were lysed in the ice-cold modified immunoprecipitation assay buffer (150 mM NaCl, 5 mM EDTA, 1% Nonidet P-40, 20 mM Tris-HCl, PH 7.5) with addition of protease and phosphatase inhibitor mixtures. 20 μg of protein from each sample was separated on 12% polyacrylamide gels and transferred to PVDF membranes. After 5% milk/TBST blotting for 1 h, primary antibodies against specific genes of interest were applied, followed by HRP-conjugated secondary antibodies. The chemiluminescent signals were detected using ECL-plus reagent (GE Healthcare). The α-tubulin detected in the same sample was used as an equal loading control. Band density analysis was performed by Image J software. All data were normalized to the band density for tubulin.

### Nitric oxide metabolite determination

Nitric oxide (NO) metabolites were detected by chemiluminescence in a Nitric Oxide Analyzer (Sievers) as described previously^58^. Briefly, 50μl of media was injected into a reaction vessel containing acetic acid and 50 mg/ml potassium iodide. This is detected by a photomultiplier tube which converts the light to an electric signal. Standard curves were performed to determine nitrite concentration, which was normalized to cell number.

### Measurement of NO production in cultured cells with DAR-1

CYGB-stimulated intracellular NO production was measured using NO-sensitive red fluorescent dye, DAR-1^59, 60^. Briefly, hCPCs with overexpressing CYGB or vector were seed in 35 mm glass bottom dishes, 10 uM DAR-1 red Dye was added 36 hours later, and cells were further maintained at 37 °C for 15 mins in dark After washing twice with PBS, cells were incubated in fresh medium for an additional 30 min to allow complete de-esterification of the intracellular diacetates. 12 fluorescence images for each group were taken by a fluorescence microscope (Leica). The intensity of DAR-1 staining was quantified using ImageJ software. Each experiment was repeated at least three times. FACS analysis for live cell staining with DAR-1 was also perfomed. Briefly, hCPCs were detached by TrypLE solution and the DAR-1 dye was then added to suspended cells with appropriate dilution (1:1000) as indicated in the protocol, and cells were further maintained at 37 °C for another one hour. After two times PBS washes to remove excess probe, replace with fresh medium and then incubate for an additional 15 min to allow complete de-esterification of the intracellular diacetates. The intensity of NO level was determined by flow cytometry analysis.

### Statistical analysis

Data were presented as means ± SEM of results taken from at least 3 independent experiments. Statistical significance was assessed by analysis of variance using Bonferroni/Dunn test, or unpaired student t-test. A *p* value less than 0.05 was considered statistically significant.

### Ethical approval and informed consent

The study was approved by the Institutional Committee on Research Involving Human Subjects in Albany Medical College (IRB#3728), and written informed consent was provided by the patients. All experiments were performed in accordance with relevant guidelines and regulations.

## Electronic supplementary material

Supplementary information
