# Supplementary material for: Cytoglobin Promotes Cardiac Progenitor Cell Survival against Oxidative Stress via the Upregulation of the NFκB/iNOS Signal Pathway and Nitric Oxide Production
Source: Sci Rep. 2017 Sep 7;7:10754. doi: 10.1038/s41598-017-11342-6 (PMC5589853; doi:10.1038/s41598-017-11342-6)
Supplement: Supplementary file 1 — Supplementary information [file 41598_2017_11342_MOESM1_ESM.pdf]

Supplementary Information

Cytoglobin Promotes Cardiac Progenitor Cell Survival against Oxidative Stress via the Upregulation of the NFκB/iNOS Signal Pathway and Nitric Oxide Production

Shuning Zhang[1,*]; Xiuchun Li[1,*]; Frances L. Jourd'heuil[1]; Shunlin Qu[1]; Neil Devijian[2]; Edward Bennett[3]; David Jourd'heuil[1, ¶]; Chuanxi Cai[1, ¶]

[1]Center for Cardiovascular Sciences, Department of Molecular and Cellular Physiology, & Department of Medicine, Albany Medical College, Albany, NY 12208, USA; [2]Division of Pediatric Cardiothoracic Surgery, Albany Medical Center, NY 12208, USA. [3]Division of Cardiothoracic Surgery, Albany Medical Center, NY 12208, USA.

*These authors contributed equally to this work.

¶Address correspondence to:
Chuanxi Cai, PhD, Center for Cardiovascular Sciences, Department of Molecular and Cellular Physiology, & Department of Medicine, Albany Medical College, Albany, NY 12208, USA. Tel.: (518) 264-2541; Fax: (518) 262-8101; Email: caic@mail.amc.edu
or
David Jourd'heuil, PhD, Department of Molecular and Cellular Physiology & Department of Medicine, Albany Medical College, Albany, NY 12208, USA. Tel.: (518) 262-8104; Fax: (518) 262-8101; Email: jourdhd@mail.amc.edu

Supplemental Figure S1. The relative expression of CYGB and myoglobin in human right atrial appendage and multiple hCPC lines. Full-length Western blot in one-gel were performed to examine the expression of CYGB and myoglobin in the human right atrial appendage (hRAA) (5 µg total protein), and multiple lines of hCPCs (20 µg total protein), which were infected with lentiviral particles expressing vector only, CYGB, scrambled shRNA, or shRNA against CYGB. ** indicates p<0.01 vs. control; n= 3 independent experiments.

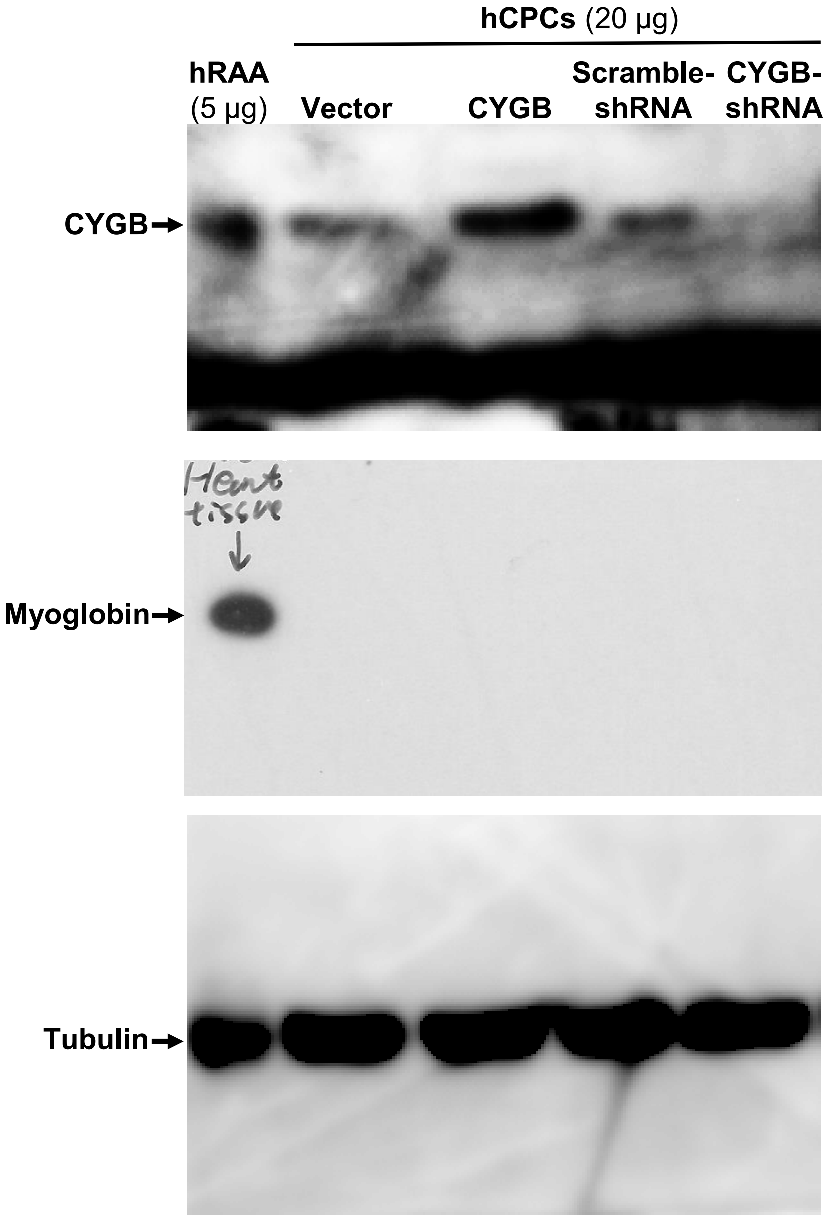

Supplemental Figure S2. The expression of CYGB is associated with the hCPC survival ability upon preconditioning with DETA-NO. (**a**) Full-length Western blot indicated CYGB was upregulated in a dose-dependent manner in response to DETA-NO. (**b**) Human CPCs were preconditioned with 250 μM of DETA-NO for 12 h, challenged with 2 mM H2O2 for 3h, and cell survival was evaluated by LDH release assay. ** indicates p<0.01 vs. vehicle control; n= 3 independent experiments.

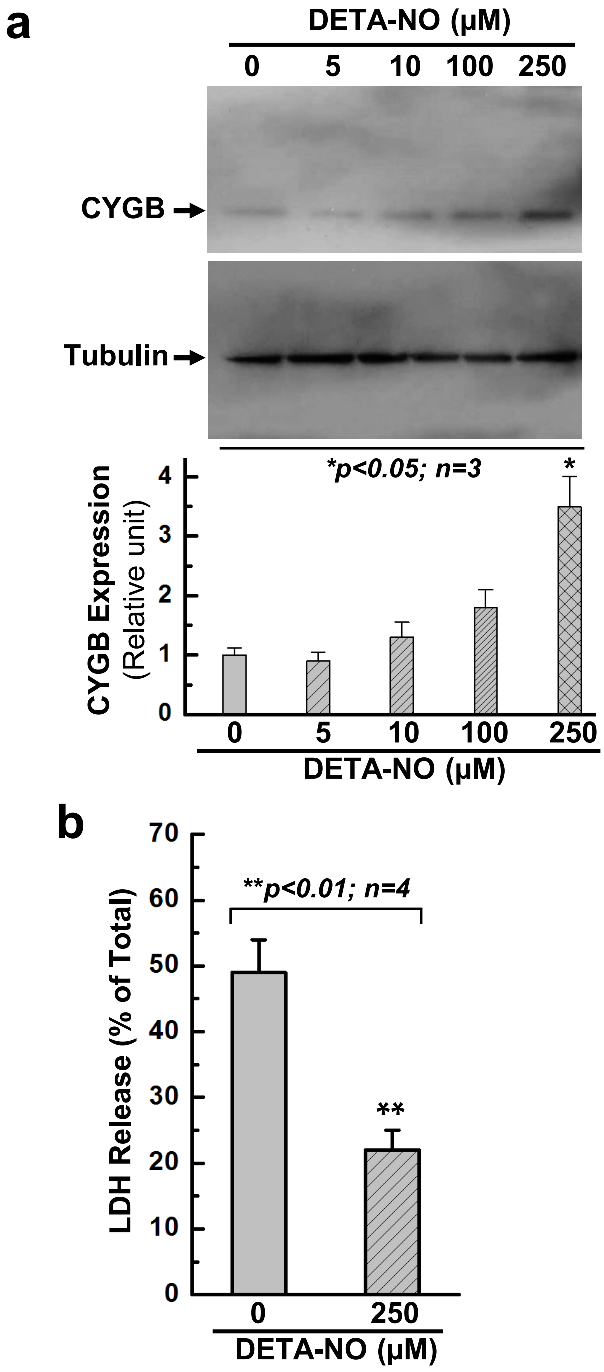

Supplemental Figure S3. Enhanced cell survival ability after overexpressing CYGB in c-kit⁺ hCPCs against the hypoxia-reoxygenation induced cell injury. (**a**) hCPCs were infected with lentivirus particles expressing either CYGB or vector only for 48 hours, challenged with 4-hour hypoxia and 2-hours reoxygenation, then evaluated by Annexin/PI FACS assay. (**b**) Quantitative data analysis for panel a. * indicates p<0.05 vs. Vector, n=3.

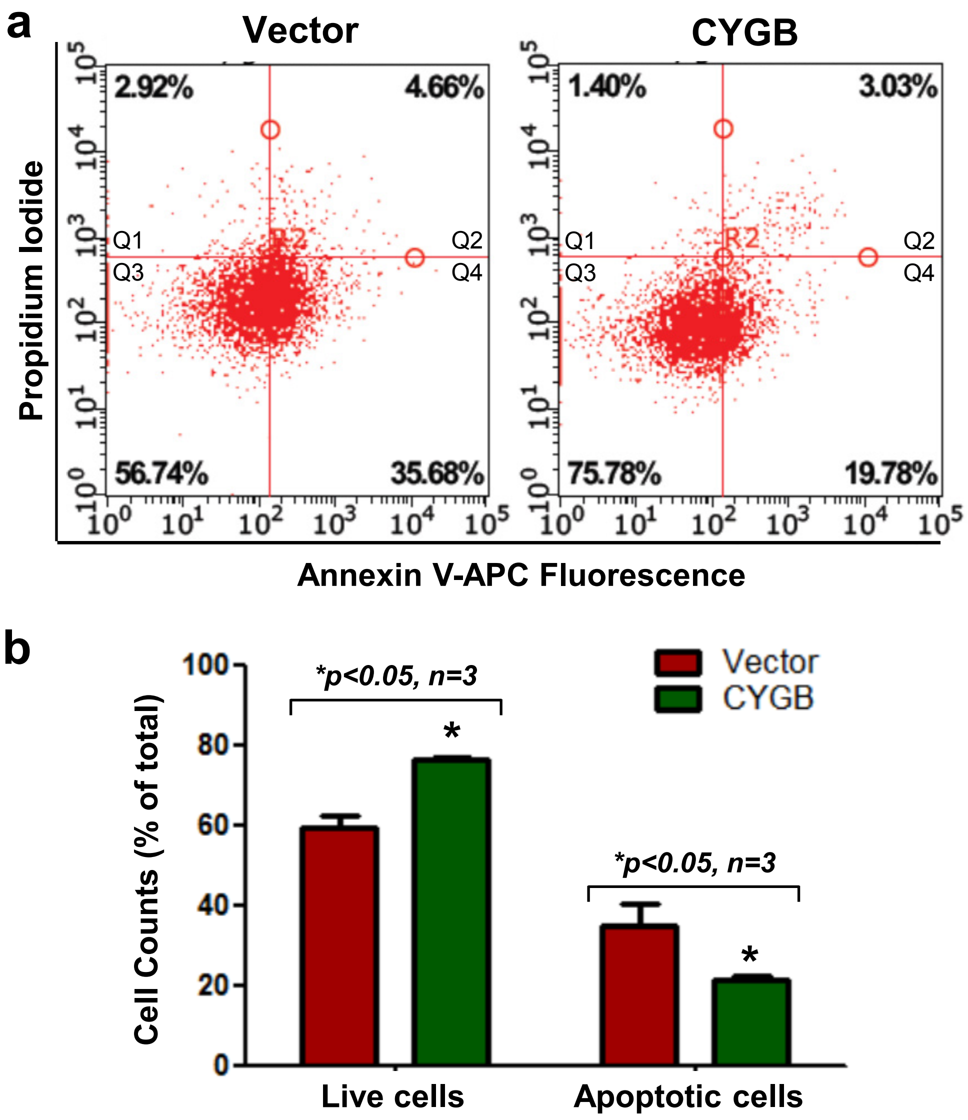

Supplemental Figure S4. Compromised cell survival ability after knocking down CYGB. (**a**) Human CPCs were infected with lentivirus particles expressing either scramble shRNA or shRNA against CYGB for 48 hours, challenged with 1 mM H_2O_2 for 1.5 h, then evaluated by Annexin/PI FACS assay. (**b**) Quantitative data analysis for panel a. ** indicates *p*<0.01 vs. control, n=6.

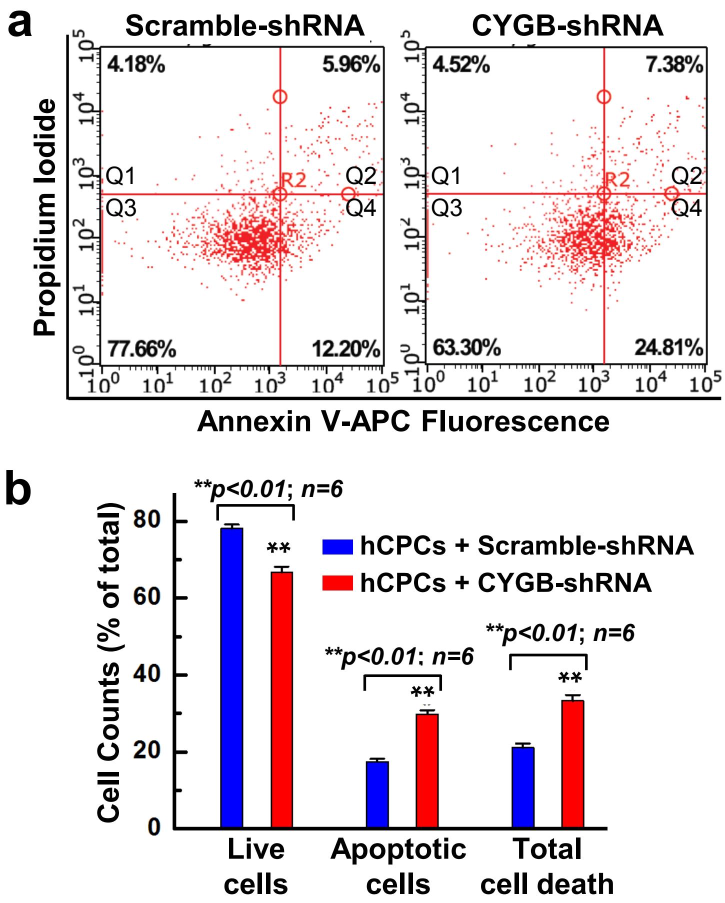

Supplemental Figure S5. Elevated cell survival ability after overexpressing CYGB in ALDH^{br}-hCPCs. (**a**) ALDH^{br}-hCPCs were infected with lentivirus particles expressing either CYGB or vector only for 48 hours, challenged with 1 mM H_2O_2 for 1.5 h, then evaluated by Annexin/PI FACS assay. (**b**) Quantitative data analysis for panel a. * indicates $p<0.05$ vs. Vector, n=3.

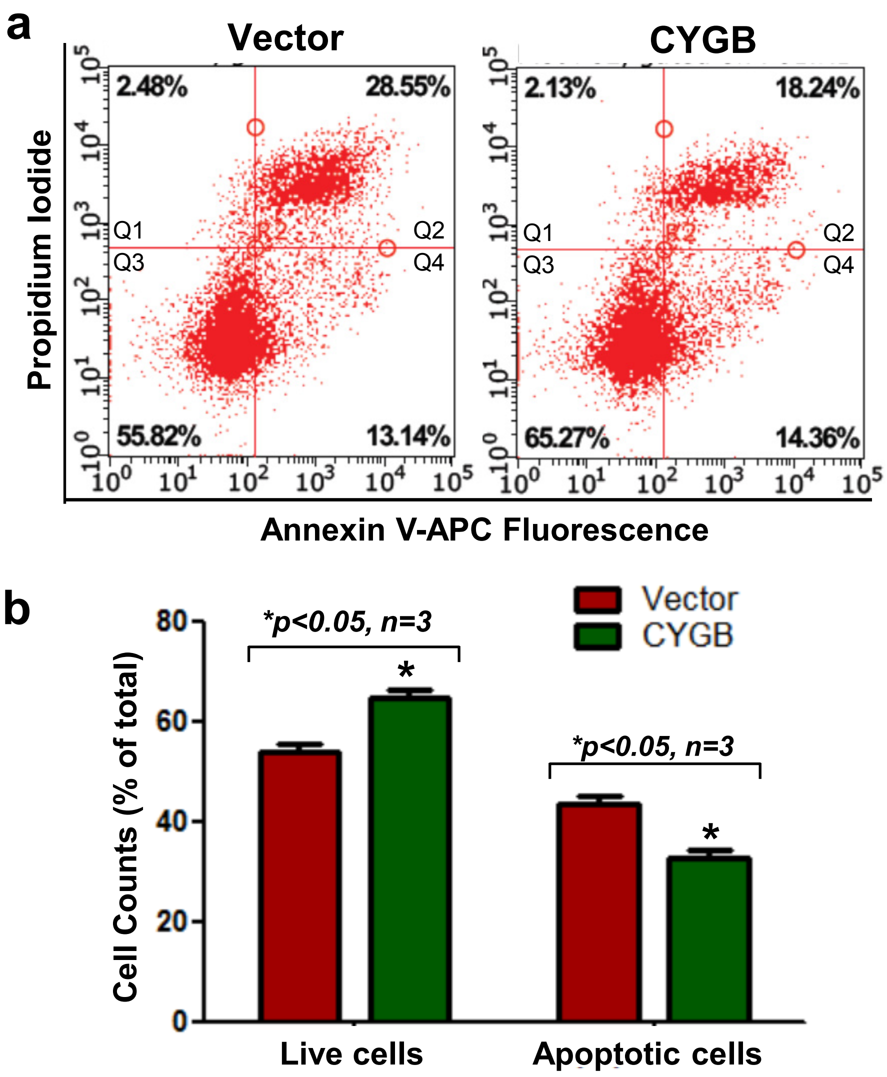

Supplemental Figure S6. Heat maps for qPCR primer library arrays. (**a**) Heat map of apoptosis library screening by a real time PCR array in response to CYGB over-expression. List of genes in this library was shown in the Supplemental Table S1. (**b**) Heat map of oxidative stress library screening by real time PCR array in response to CYGB overexpression. List of genes in this library was shown in the Supplemental Table S2.

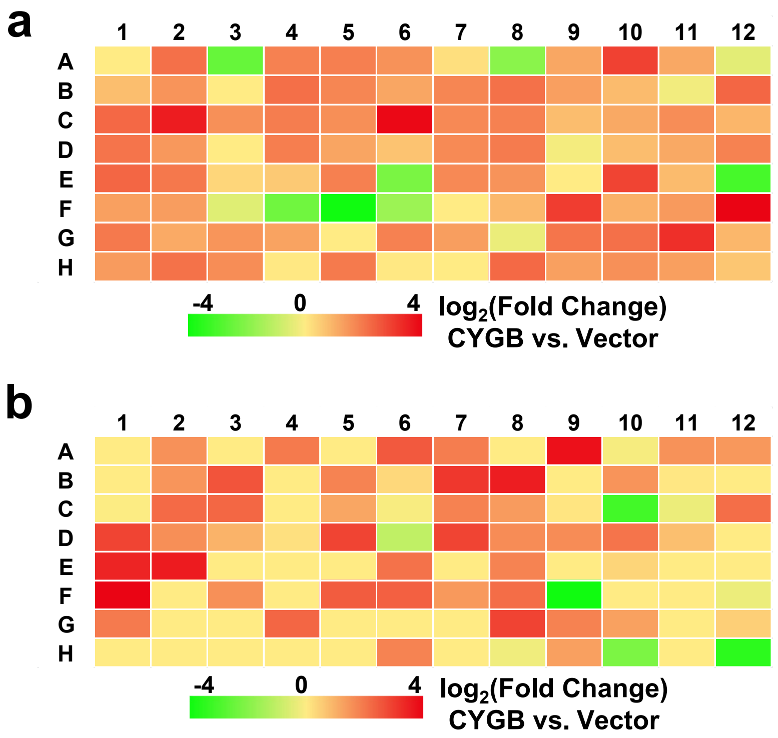

Supplemental Figure S7. Overexpressing CYGB did not affect the expression of cardiac lineage genes in hCPCs. Examination of basal gene expression of *GATA4* (**a**), *NKX2.5* (**b**), *MEF2C* (**c**) and *TBX5* (**d**) at the mRNA level after CYGB was overexpressed. *N.S.* indicates no significant difference between two groups of cells.

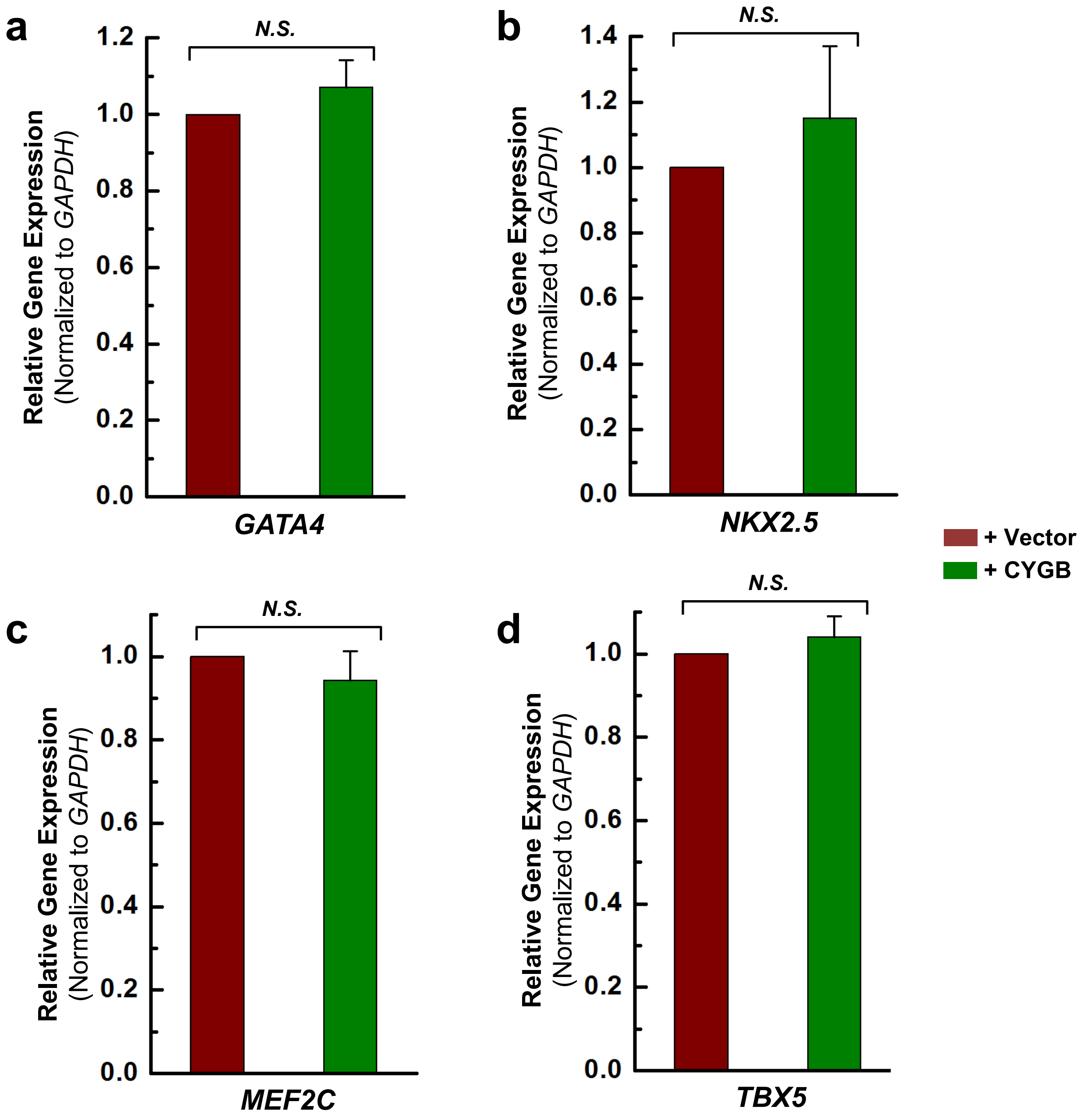

Supplemental Figure S8. The full-length blot for CYGB and Tubulin in Figure 1d.

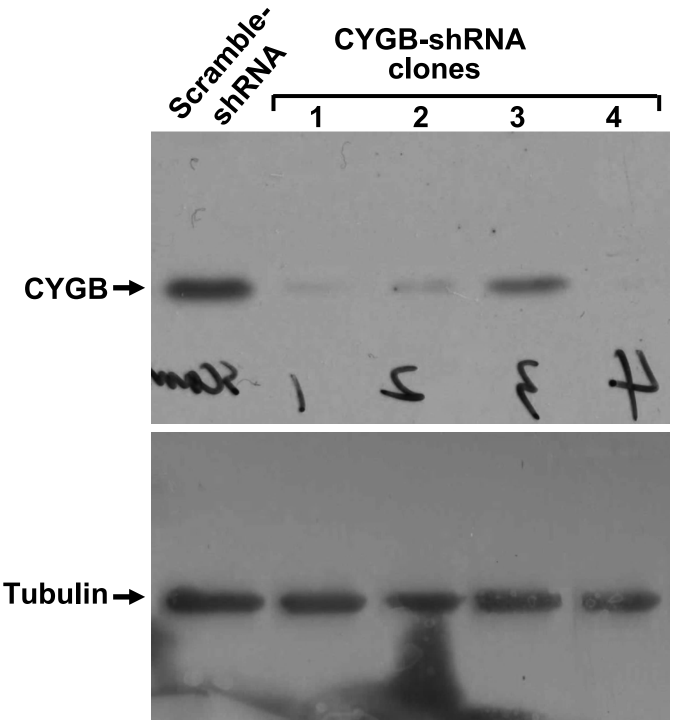

Supplemental Figure S9. The expression of eNOS and nNOS are not present in hCPCs. (**a**) Full-length Western blot indicated eNOS was expressed in HUVEC cells, but not detected in hCPCs with or without CYGB overexpression. (**b**) Full-length Western blot indicated nNOS was not expressed in hCPCs with or without CYGB overexpression; lysates from human brain tissue purchased from National Disease Research Interchange (NDRI) serve as the positive control. n= 3 independent experiments.

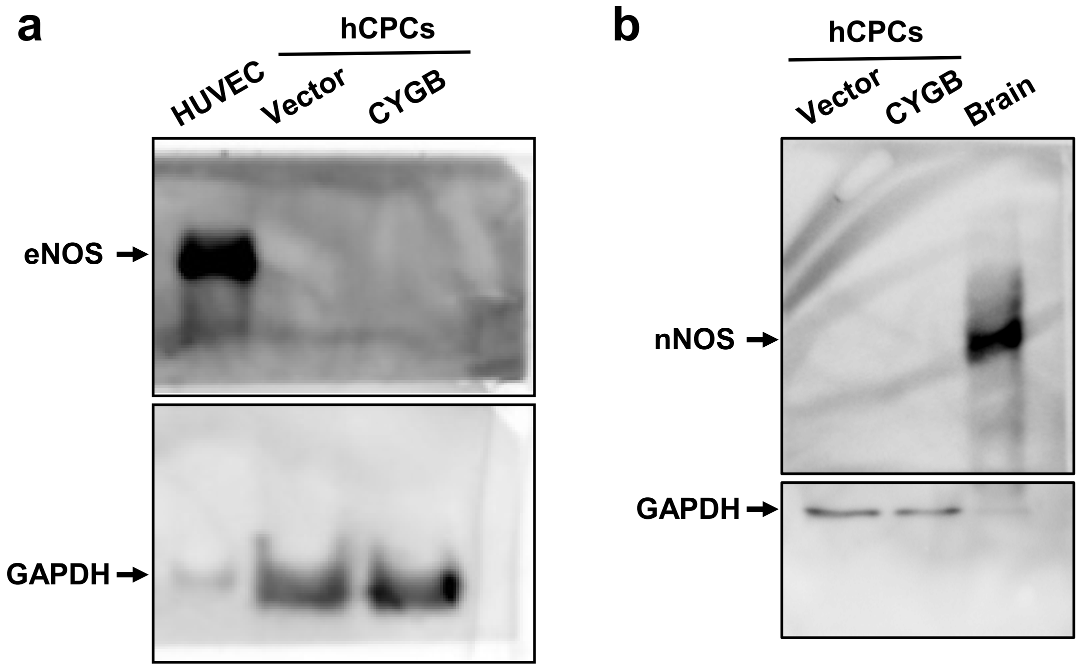

Supplemental Figure S10. The full-length blots for BCL2, BCL-xL, MCL1, TRAF1, TRAF5, TRAF4, CRADD and Tubulin in Figure 2e.

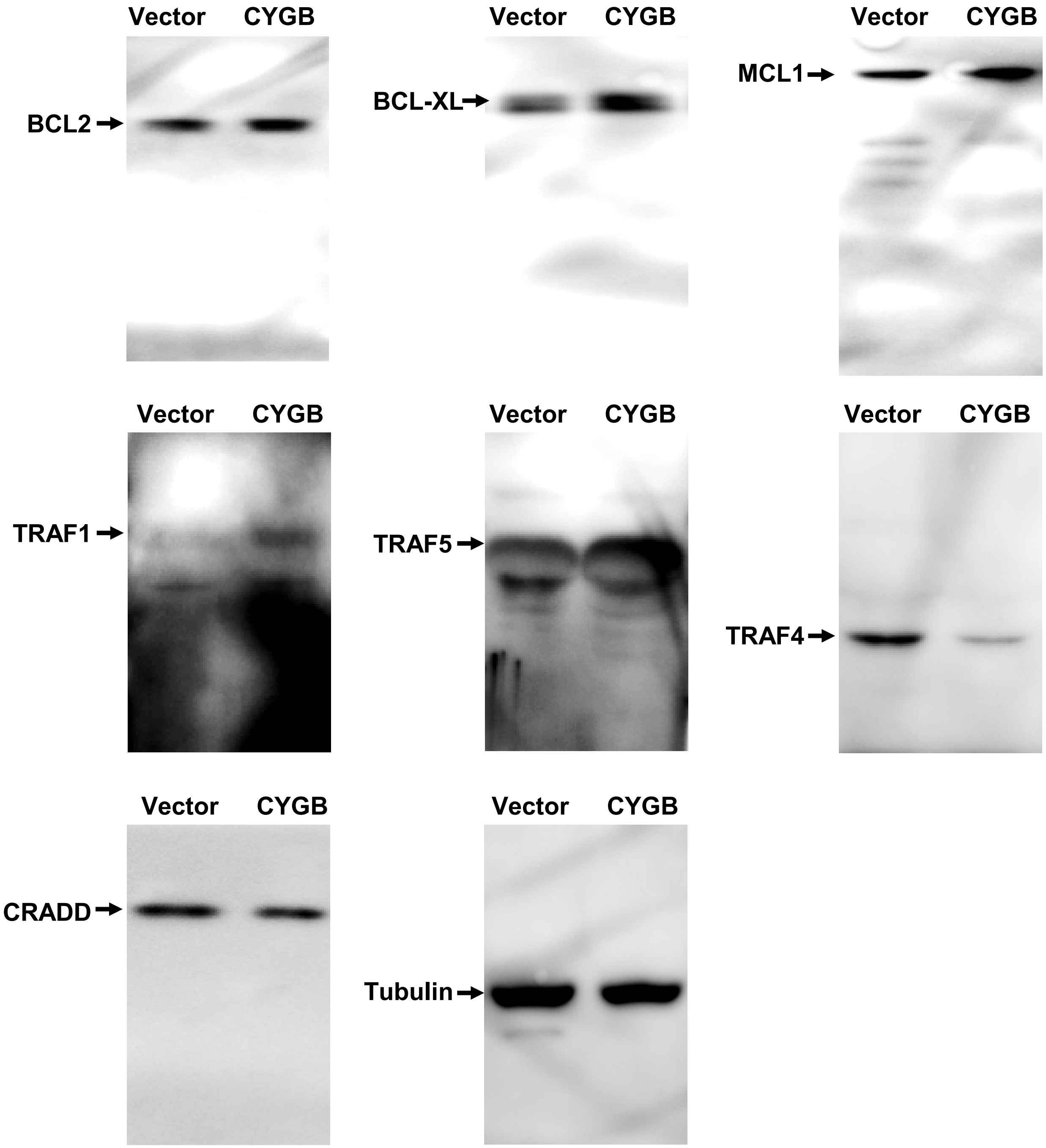

Supplemental Figure S11. The full-length blot for CYGB, PRDX1, SOD2 and Tubulin in Figure 3f.

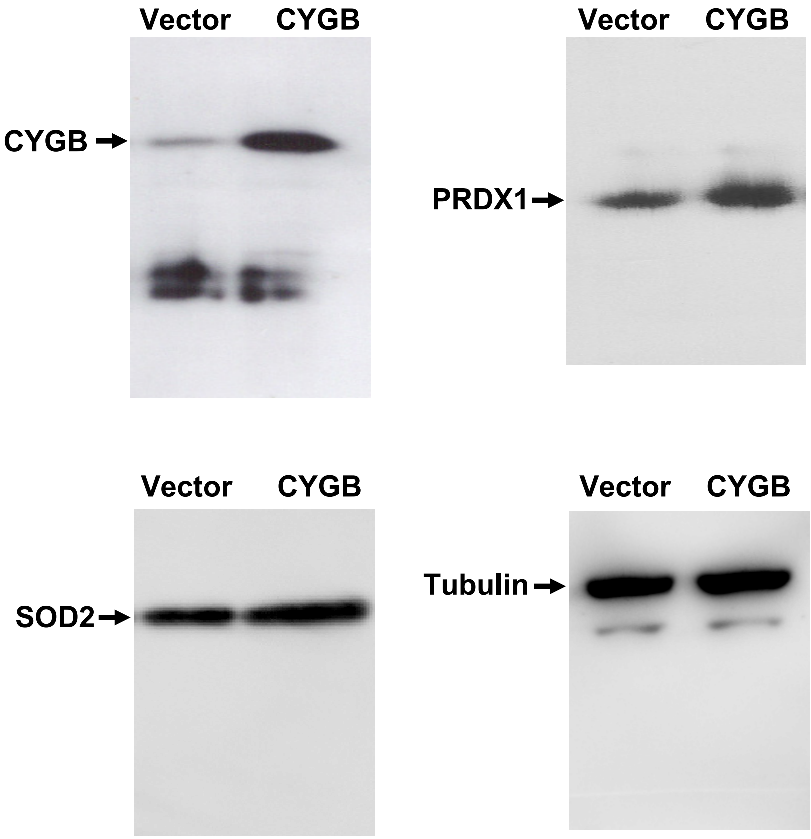

Supplemental Figure S12. The full-length blot for HMOX1, IKBKB, TLR1, TLR4, NFkB-p65, and Tubulin in Figure 4c.

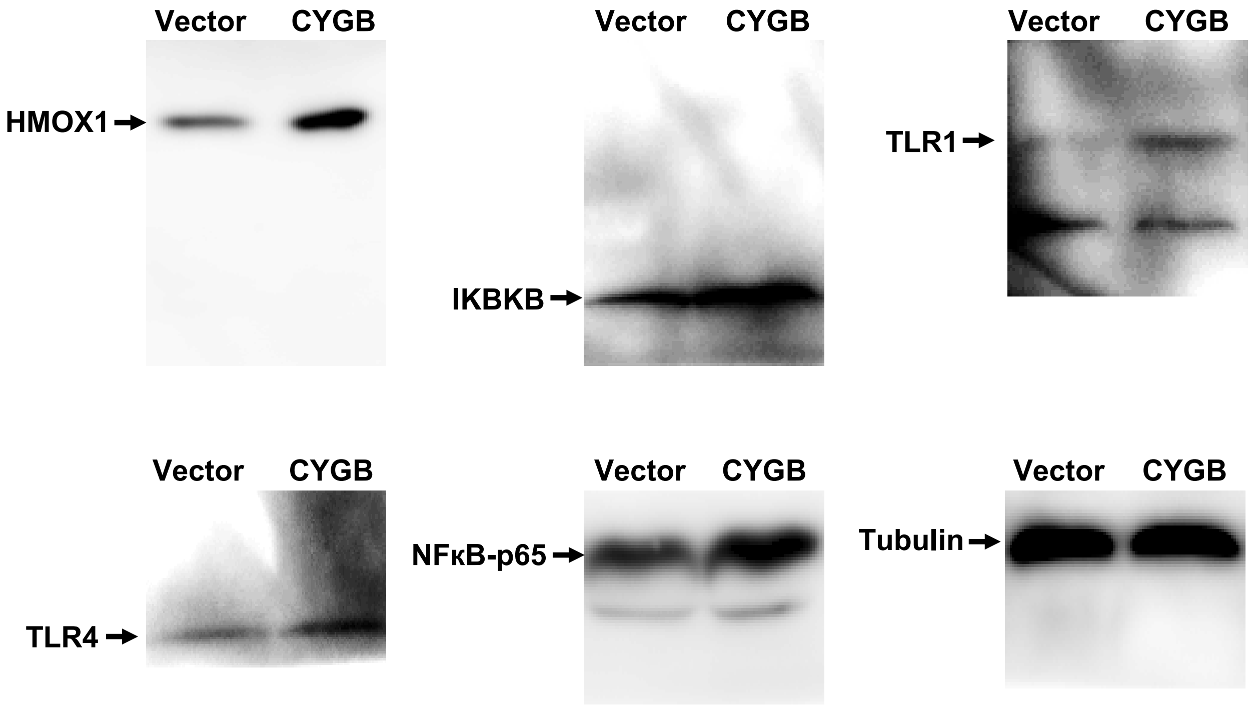

Supplemental Figure S13. The full-length blot for NFκB-p65, IKBKB, iNOS, HMOX1, TLR4, and Tubulin in Figure 5c.

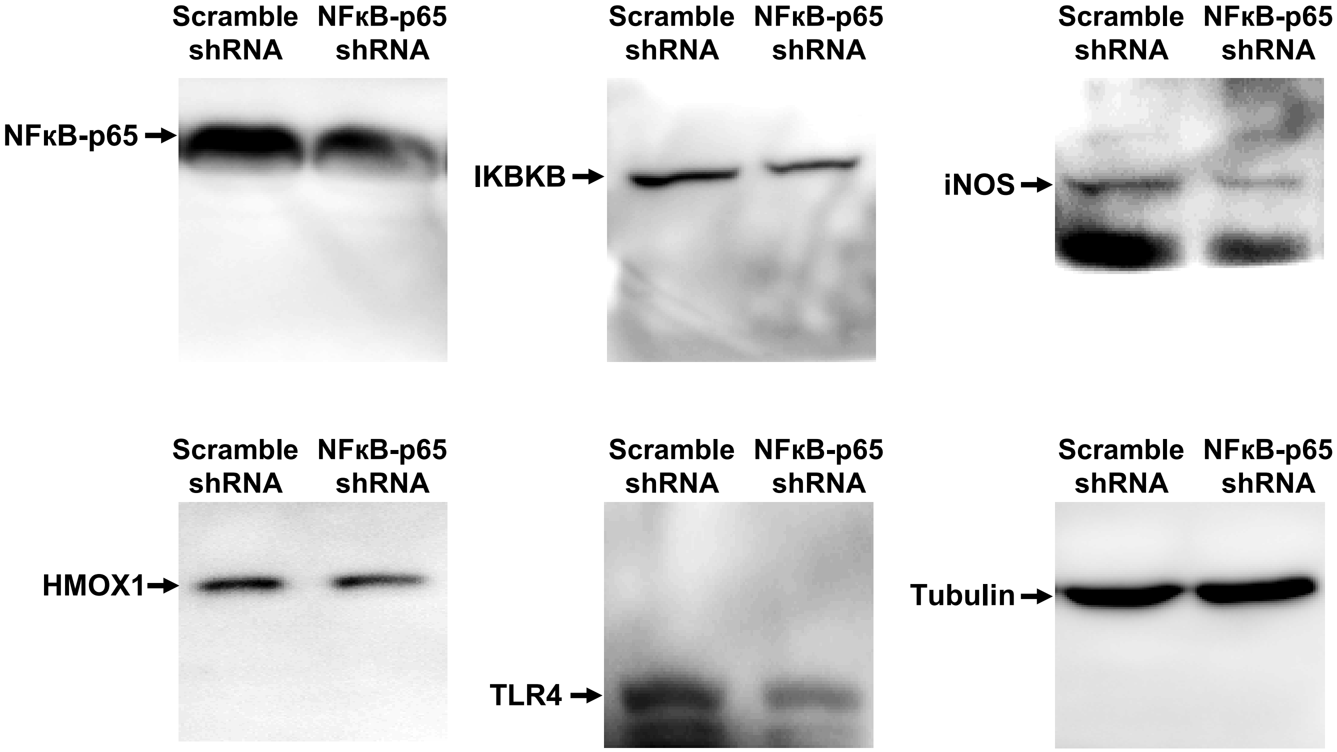

Supplemental Figure S14. The full-length blot for BCL2, BCL-XL, MCL1, CYGB, SOD2, and Tubulin in Figure 5d.

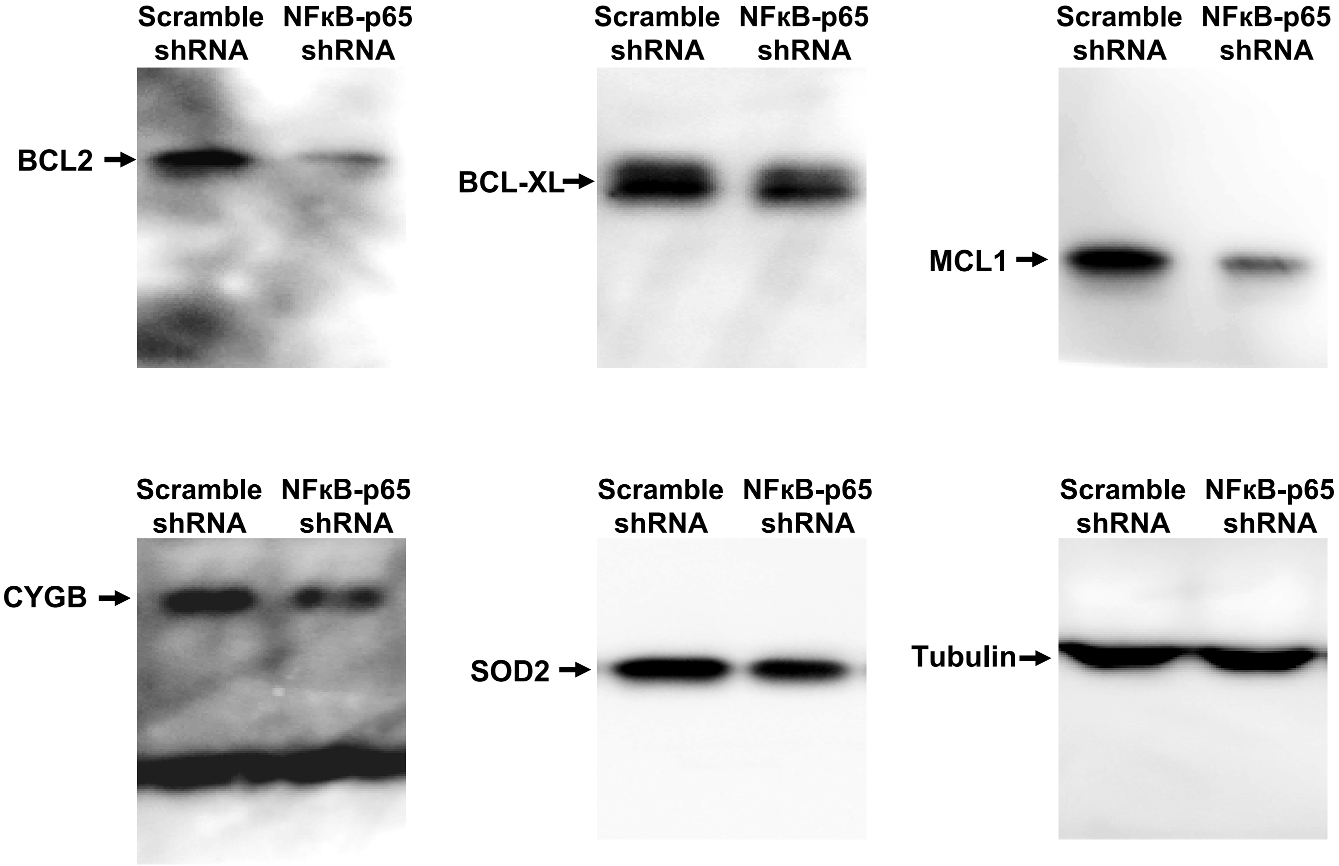

Supplemental Figure S15. The full-length blot for iNOS and Tubulin in Figure 6a.

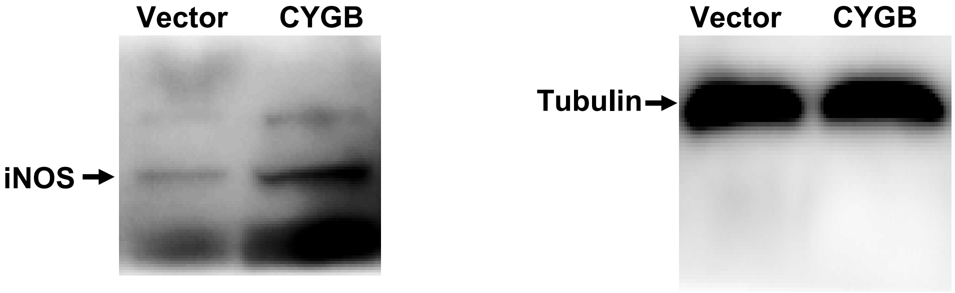

Supplemental Figure S16. The full-length blot for iNOS, BCL2, BCL-XL, MCL1, CYGB, SOD2, HMOX1, TLR4, NFkB-p65, IKBKB, and Tubulin in Figure 7c.

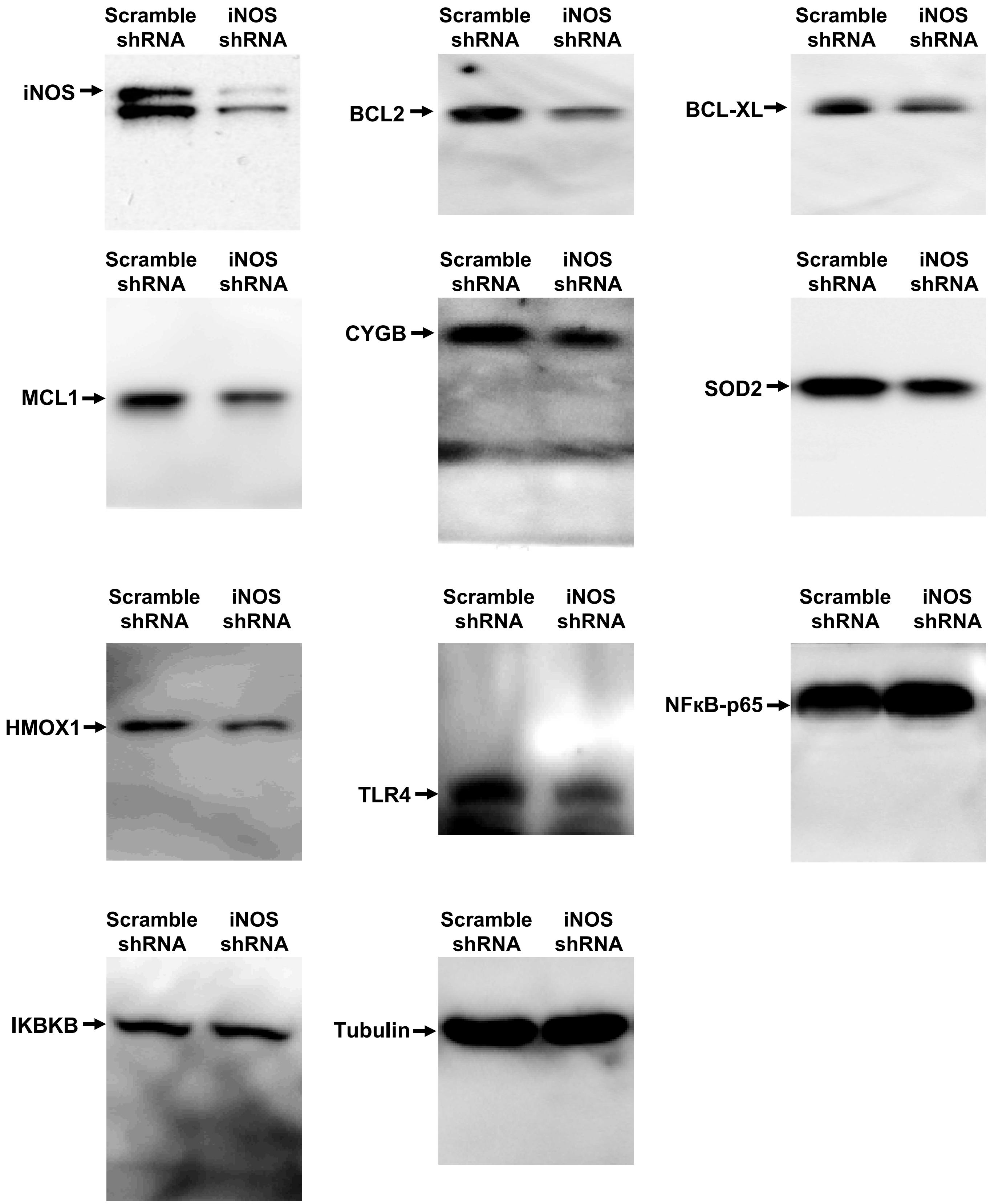

Supplemental Table S1. Gene list for the human apoptosis primer library. This primer library contains 88 primer sets directed against apoptosis genes and 8 housekeeping gene primer sets. Adapted from: http://www.realtimeprimers.com/huapprli.html.

well	Symbol	Name	well	Symbol	Name
A1	APAF1	Apoptotic protease activating factor	E1	MCL1	Myeloid cell leukemia sequence 1 (BCL2-related)
A2	ATM	Ataxia telangiectasia mutated	E2	MYD88	Myeloid differentiation primary response gene (88)
A3	BAG1	BCL2-associated athanogene	E3	CARD4	Caspase recruitment domain family, member 4
A4	BAG3	BCL2-associated athanogene 3	E4	FAS	Fas (TNF receptor superfamily, member 6)
A5	BAG4	BCL2-associated athanogene 4	E5	RIPK2	Receptor-interacting serine-threonine kinase 2
A6	BAK1	BCL2-antagonist/killer 1	E6	RPA3	Replication protein A3, 14kDa
A7	BAX	BCL2-associated X protein	E7	TANK	TRAF family member-associated NFKB activator
A8	BCL10	B-cell CLL/lymphoma 10	E8	TNF	Tumor necrosis factor (TNF superfamily, member 2)
A9	BCL2	B-cell CLL/lymphoma 2	E9	TNFRSF10A	Tumor necrosis factor receptor superfamily, member 10a
A10	BCL2A1	BCL2-related protein A1	E10	TNFRSF10B	Tumor necrosis factor receptor superfamily, member 10b
A11	BCL2L1	BCL2-like 1	E11	TNFRSF10C	Tumor necrosis factor receptor superfamily, member 10c
A12	BCL2L11	BCL2-like 11 (apoptosis facilitator)	E12	TNFRSF10D	Tumor necrosis factor receptor superfamily, member 10d
B1	BCL2L2	BCL2-like 2	F1	TNFRSF11B	Tumor necrosis factor receptor superfamily, member 11b
B2	BFAR	Bifunctional apoptosis regulator	F2	TNFRSF17	Tumor necrosis factor receptor superfamily, member 17
B3	BIK	BCL2-interacting killer (apoptosis-inducing)	F3	TNFRSF1A	Tumor necrosis factor receptor superfamily, member 1A
B4	BIRC1	Baculoviral IAP repeat-containing 1	F4	TNFRSF21	Tumor necrosis factor receptor superfamily, member 21
B5	BIRC2	Baculoviral IAP repeat-containing 2	F5	CD40	CD40 antigen (TNF receptor superfamily member 5)
B6	BIRC3	Baculoviral IAP repeat-containing 3	F6	TNFRSF8	Tumor necrosis factor receptor superfamily, member 8
B7	BIRC4	Baculoviral IAP repeat-containing 4	F7	TNFRSF9	Tumor necrosis factor receptor superfamily, member 9
B8	BIRC5	Baculoviral IAP repeat-containing 5 (survivin)	F8	TNFSF10	Tumor necrosis factor (ligand) superfamily, member 10
B9	BIRC6	Baculoviral IAP repeat-containing 6 (apollon)	F9	TNFSF11	Tumor necrosis factor (ligand) superfamily, member 11
B10	BNIP3	BCL2/adenovirus E1B 19kDa interacting protein 3	F10	TNFSF13	Tumor necrosis factor (ligand) superfamily, member 12
B11	BRE	Brain and reproductive organ-expressed	F11	TNFSF13B	Tumor necrosis factor (ligand) superfamily, member 13b
B12	BOK	BCL2-related ovarian killer	F12	TNFSF15	Tumor necrosis factor (ligand) superfamily, member 15
C1	CASP1	Caspase 1, apoptosis-related cysteine protease	G1	TNFSF18	Tumor necrosis factor (ligand) superfamily, member 18
C2	CASP10	Caspase 10, apoptosis-related cysteine protease	G2	TNFSF4	Tumor necrosis factor (ligand) superfamily, member 4
C3	CASP4	Caspase 4, apoptosis-related cysteine peptidase	G3	CD40LG	CD40 ligand (TNF superfamily, member 5)
C4	CASP2	Caspase 2, apoptosis-related cysteine protease	G4	FASLG	Fas ligand (TNF superfamily, member 6)
C5	CASP3	Caspase 3, apoptosis-related cysteine protease	G5	TNFSF7	Tumor necrosis factor (ligand) superfamily, member 7
C6	CASP5	Caspase 5, apoptosis-related cysteine protease	G6	TNFSF8	Tumor necrosis factor (ligand) superfamily, member 8
C7	CASP6	Caspase 6, apoptosis-related cysteine protease	G7	TNFSF9	Tumor necrosis factor (ligand) superfamily, member 9
C8	CASP7	Caspase 7, apoptosis-related cysteine protease	G8	TP53	Tumor protein p53 (Li-Fraumeni syndrome)
C9	CASP8	Caspase 8, apoptosis-related cysteine protease	G9	TP73L	Tumor protein p73-like
C10	CASP8AP2	CASP8 associated protein 2	G10	TRAF1	TNF receptor-associated factor 1
C11	CFLAR	CASP8 and FADD-like apoptosis regulator	G11	TRAF2	TNF receptor-associated factor 2
C12	CHEK1	CHK1 checkpoint homolog (S. pombe)	G12	TRAF3	TNF receptor-associated factor 3
D1	CHEK2	CHK2 checkpoint homolog (S. pombe)	H1	TRAF4	TNF receptor-associated factor 4
D2	CIDEA	Cell death-inducing DFFA-like effector a	H2	TRAF5	TNF receptor-associated factor 5
D3	CIDEB	Cell death-inducing DFFA-like effector b	H3	TRAF6	TNF receptor-associated factor 6
D4	CRADD	CASP2 and RIPK1 domain containing adaptor	H4	TRIP	TRAF interacting protein
D5	DAPK1	Death-associated protein kinase 1	H5	ACTB	Actin, beta
D6	DAPK2	Death-associated protein kinase 2	H6	B2M	Beta-2-microglobulin
D7	DFFA	DNA fragmentation factor, 45kDa, alpha polypeptide	H7	GAPD	Glyceraldehyde-3-phosphate dehydrogenase
D8	DFFB	DNA fragmentation factor, 40kDa, beta polypeptide	H8	GUSB	Glucuronidase, beta
D9	FADD	Fas (TNFRSF6)-associated via death domain	H9	HPRT1	Hypoxanthine phosphoribosyltransferase 1
D10	GADD45A	Growth arrest and DNA-damage-inducible, alpha	H10	PGK	Phosphoglycerate kinase 1
D11	HRK	Harakiri, BCL2 interacting protein	H11	PPIA	Peptidylprolyl isomerase A
D12	LTBR	Lymphotoxin beta receptor (TNFR superfamily, member 3	H12	RPL13A	Ribosomal protein L13a

Supplemental Table S2. **Gene list for the human oxidative stress primer library.**

This primer library contains 88 primer sets directed against oxidative stress genes and 8 housekeeping gene primer sets. Adapted from: http://realtimeprimers.com/huoxstprli.html.

well	Symbol	Name	well	Symbol	Name
A1	ALB	Albumin	E1	NCF2	Neutrophil cytosolic factor 2
A2	ALOX12	Arachidonate 12-lipoxygenase	E2	NME5	Non-metastatic cells 5
A3	ANGPTL7	Angiopoietin-like 7	E3	NOS2	Nitric oxide synthase 2
A4	AOX1	Aldehyde oxidase 1	E4	NOS2A	Nitric oxide synthase 2A
A5	APOE	Apolipoprotein E	E5	NOX5	NADPH oxidase, EF-hand calcium binding domain 5
A6	ATOX1	ATX1 antioxidant protein 1 homolog (yeast)	E6	NUDT1	Nudix -type motif 1
A7	BNIP3	BCL2/adenovirus E1B 19kDa interacting protein 3	E7	NUDT2	Nudix -type motif 2
A8	CAT	Catalase	E8	OXR1	Oxidation resistance 1
A9	CSDE1	Cold shock domain containing E1, RNA-binding	E9	OXSR1	Oxidative-stress responsive 1
A10	CYBA	Cytochrome b-245, alpha polypeptide	E10	PDLIM1	PDZ and LIM domain 1 (elfin)
A11	CYGB	Cytoglobin	E11	PIP3-E	Phosphoinositide-binding protein PIP3-E
A12	DHCR24	24-dehydrocholesterol reductase	E12	PNKP	Polynucleotide kinase 3'-phosphatase
B1	DUOX1	Dual oxidase 1	F1	PRDX1	Peroxiredoxin 1
B2	DUOX2	Dual oxidase 2	F2	PRDX2	Peroxiredoxin 2
B3	DUSP1	Dual specificity phosphatase 1	F3	PRDX3	Peroxiredoxin 3
B4	EPHX2	Epoxide hydrolase 2, cytoplasmic	F4	PRDX4	Peroxiredoxin 4
B5	EPX	Eosinophil peroxidase	F5	PRDX5	Peroxiredoxin 5
B6	FOXM1	Forkhead box M1	F6	PRDX6	Peroxiredoxin 6
B7	GLRX	Glutaredoxin	F7	PREX1	Phosphatidylinositol 3,4,5-trisphosphate-dependent RAC exchanger 1
B8	GLRX2	Glutaredoxin 2	F8	PRG3	Proteoglycan 3
B9	GPR156	G protein-coupled receptor 156	F9	PRNP	Prion protein (p27-30)
B10	GPX1	Glutathione peroxidase 1	F10	PTGS1	Prostaglandin-endoperoxide synthase 1
B11	GPX2	Glutathione peroxidase 2	F11	PTGS2	Prostaglandin-endoperoxide synthase 2
B12	GPX3	Glutathione peroxidase 3	F12	PXDN	Peroxidasin homolog
C1	GPX4	Glutathione peroxidase 4	G1	PXDNL	Peroxidasin homolog (Drosophila)-like
C2	GPX5	Glutathione peroxidase 5	G2	RNF7	Ring finger protein 7
C3	GPX6	Glutathione peroxidase 6	G3	SCARA3	Scavenger receptor class A, member 3
C4	GPX7	Glutathione peroxidase 7	G4	SEPP1	Selenoprotein P, plasma, 1
C5	GSR	Glutathione reductase	G5	SFTPD	Surfactant, pulmonary-associated protein D
C6	GSS	Glutathione synthetase	G6	SGK2	Serum/glucocorticoid regulated kinase 2
C7	GSTA4	Glutathione S-transferase A4	G7	SIRT2	Sirtuin 2
C8	GSTM2	Glutathione S-transferase M2 (muscle)	G8	SOD1	Superoxide dismutase 1, soluble
C9	GSTM3	Glutathione S-transferase M3 (brain)	G9	SOD2	Superoxide dismutase 2, mitochondrial
C10	GSTM5	Glutathione S-transferase mu 5	G10	SOD3	Superoxide dismutase 3, extracellular
C11	GSTP1	Glutathione S-transferase pi	G11	SRXN1	Sulfiredoxin 1 homolog (S. cerevisiae)
C12	GSTT1	Glutathione S-transferase theta 1	G12	STK25	Serine/threonine kinase 25 (STE20 homolog, yeast)
D1	GSTZ1	Glutathione transferase zeta 1	H1	TPO	Thyroid peroxidase
D2	GTF2I	General transcription factor II, i	H2	TTN	Titin
D3	KRT1	Keratin 1 (epidermolytic hyperkeratosis)	H3	TXNRD1	Thioredoxin reductase 1
D4	LPO	Lactoperoxidase	H4	TXNRD2	Thioredoxin reductase 2
D5	MBL2	Mannose-binding lectin (protein C) 2, soluble	H5	ACTB	Actin, beta
D6	MGST3	Microsomal glutathione S-transferase 3	H6	B2M	Beta-2-microglobulin
D7	MPV17	MpV17 mitochondrial inner membrane protein	H7	GAPD	Glyceraldehyde-3-phosphate dehydrogenase
D8	MSRA	Methionine sulfoxide reductase A	H8	GUSB	Glucuronidase, beta
D9	MT2A	Metallothionein 2A	H9	HPRT1	Hypoxanthine phosphoribosyltransferase 1
D10	MT3	Metallothionein 3	H10	PGK	Phosphoglycerate kinase 1
D11	MTL5	Metallothionein-like 5, testis-specific (tesmin)	H11	PPIA	Peptidylprolyl isomerase A
D12	NCF1	Neutrophil cytosolic factor 1	H12	RPL13A	Ribosomal protein L13a

Supplemental table S3. Gene list for the human NFKappaB (NFκB) primer library.

This primer library contains 88 primer sets directed against NFKappaB signaling genes and 8 housekeeping gene primer sets. Adapted from: http://realtimeprimers.com/hunfprli.html.

well	Symbol	Name	well	Symbol	Name
A1	AGT	Angiotensinogen	E1	LTB	Lymphotoxin beta (TNF superfamily, member 3)
A2	AKT1	V-akt murine thymoma viral oncogene homolog 1	E2	LTBR	Lymphotoxin beta receptor
A3	ATF1	Activating transcription factor 1	E3	MALT1	Mucosa associated lymphoid tissue lymphoma translocation gene 1
A4	BCL10	B-cell CLL/lymphoma 10	E4	MAP3K1	Mitogen-activated protein kinase kinase kinase 1
A5	BCL2	B-cell CLL/lymphoma 2	E5	MMP7	Matrix metallopeptidase 7 (matrilysin, uterine)
A6	BCL2L1	BCL2-like 1	E6	MMP9	Matrix metallopeptidase 9
A7	BCL3	B-cell CLL/lymphoma 3	E7	NFKB1	Nuclear factor of kappa light polypeptide gene enhancer in B-cells 1 (p105)
A8	BIRC2	Baculoviral IAP repeat-containing 2	E8	NFKB2	Nuclear factor of kappa light polypeptide gene enhancer in B-cells 2 (p49/p100)
A9	BIRC4	Baculoviral IAP repeat-containing 4	E9	NFKBIA	Nuclear factor of kappa light polypeptide gene enhancer in B-cells inhibitor, alpha
A10	CASP1	Caspase 1, apoptosis-related cysteine peptidase	E10	NLRP12	NLR family, pyrin domain containing 12
A11	CASP8	apoptosis-related cysteine peptidase	E11	NOD1	Nucleotide-binding oligomerization domain containing 1
A12	CCL2	Chemokine (C-C motif) ligand 2	E12	PPM1A	Protein phosphatase 1A (formerly 2C), alpha isoform
B1	CD40	CD40 molecule, TNF receptor superfamily member 5	F1	RAF1	V-raf-1 murine leukemia viral oncogene homolog 1
B2	CFLAR	CASP8 and FADD-like apoptosis regulator	F2	REL	V-rel reticuloendotheliosis viral oncogene homolog (avian)
B3	CHUK	Conserved helix-loop-helix ubiquitous kinase	F3	RELA	V-rel reticuloendotheliosis viral oncogene homolog A
B4	CSF1	Colony stimulating factor 1 (macrophage)	F4	RELB	V-rel reticuloendotheliosis viral oncogene homolog B
B5	CSF2	Colony stimulating factor 2 (granulocyte- macrophage)	F5	RHOA	Ras homolog gene family, member A
B6	CSF3	Colony stimulating factor 3 (granulocyte)	F6	RIPK1	Receptor (TNFRSF)-interacting serine-threonine kinase 1
B7	EDARADD	EDAR-associated death domain	F7	SELL	Selectin L
B8	EDG2	Lysophosphatidic acid receptor 1	F8	SELP	Selectin P
B9	EGR1	Early growth response 1	F9	STAT1	Signal transducer and activator of transcription 1
B10	ELK1	ELK1, member of ETS oncogene family	F10	TBK1	TANK binding kinase 1
B11	F2R	Coagulation factor II (thrombin) receptor	F11	TICAM1	Toll-like receptor adaptor molecule 1
B12	FADD	Fas (TNFRSF6)-associated via death domain	F12	TICAM2	Toll-like receptor adaptor molecule 2
C1	FASLG	Fas ligand (TNF superfamily, member 6)	G1	TLR1	Toll-like receptor 1
C2	FOS	V-fos FBJ murine osteosarcoma viral oncogene	G2	TLR2	Toll-like receptor 2
C3	GJA1	Gap junction protein, alpha 1, 43kDa	G3	TLR3	Toll-like receptor 3
C4	HMOX1	Heme oxygenase (decycling) 1	G4	TLR4	Toll-like receptor 4
C5	HTR2B	5-hydroxytryptamine (serotonin) receptor 2B	G5	TLR7	Toll-like receptor 7
C6	ICAM1	Intercellular adhesion molecule 1 (CD54)	G6	TLR8	Toll-like receptor 8
C7	IFNA1	Interferon, alpha 1	G7	TLR9	Toll-like receptor 9
C8	IFNAB1	Interferon, beta 1	G8	TMED4	Transmembrane emp24 protein transport domain containing 4
C9	IFNG	Interferon, gamma	G9	TNF	Tumor necrosis factor (TNF superfamily, member 2)
C10	IKBKB	Inhibitor of kappa light polypeptide gene enhancer in B-cells, kinase beta	G10	TNFAIP3	Tumor necrosis factor, alpha-induced protein 3
C11	IKBKE	Inhibitor of kappa light polypeptide gene enhancer in B-cells, kinase epsilon	G11	TNFRSF10A	Tumor necrosis factor receptor superfamily, member 10a
C12	IKBKG	Inhibitor of kappa light polypeptide gene enhancer in B-cells, kinase gamma	G12	TNFRSF10B	Tumor necrosis factor receptor superfamily, member 10b
D1	IL10	Interleukin 10	H1	TNFRSF1A	Tumor necrosis factor receptor superfamily, member 1A
D2	IL12A	Interleukin 12A	H2	TNFSF10	Tumor necrosis factor (ligand) superfamily, member 10
D3	IL12B	Interleukin 12B	H3	TNFSF15	Tumor necrosis factor (ligand) superfamily, member 15
D4	IL1A	Interleukin 1, alpha	H4	TRADD	TNFRSF1A-associated via death domain
D5	IL1B	Interleukin 1, beta	H5	ACTB	Actin, beta
D6	IL1R1	Interleukin 1 receptor, type I	H6	B2M	Beta-2-microglobulin
D7	IL6	Interleukin 6 (interferon, beta 2)	H7	GAPD	Glyceraldehyde-3-phosphate dehydrogenase
D8	IL8	Interleukin 8	H8	GUSB	Glucuronidase, beta
D9	IRAK1	Interleukin-1 receptor-associated kinase 1	H9	HPRT1	Hypoxanthine phosphoribosyltransferase 1
D10	IRAK2	Interleukin-1 receptor-associated kinase 2	H10	PGK1	Phosphoglycerate kinase 1
D11	JUN	Jun oncogene	H11	PPIA	Peptidylprolyl isomerase A
D12	LTA	Lymphotoxin alpha (TNF superfamily, member 1)	H12	RPL13A	Ribosomal protein L13a

Supplemental table S4. **List of the antibodies used in immunoblot analysis.**

Antigen	Source	Catalog No.	Ratio for dilution
CYGB	Miami Valley Biotech	CAB01	1:500
PRDX1	R&D Systems	AF3488	1:10000
TRAF1	R&D Systems	AF3276	1:500
TRAF4	R&D Systems	AF3279	1:500
CRADD	R&D Systems	AF4680	1:500
IKBKB	R&D Systems	AF4535	1:500
TLR1	R&D Systems	AF1484	1:1000
TLR4	R&D Systems	AF1478	1:1000
SOD2	Santa Cruz	sc-130345	1:10000
HMOX-1	Santa Cruz	sc-136960	1:2000
BCL2	Cell Signaling	2870	1:1000
BCL-xL	Cell Signaling	2764	1:1000
MCL-1	Cell Signaling	5453	1:1000
NFκB-p65	Cell Signaling	8242	1:1000
TRAF5	Enzo Life Sciences, Inc	ALX-804-250-C100	1:1000
CYGB	Miami Valley Biotech	CAB01	1:500
iNOS	Abcam	ab3523	1:1000
eNOS	R&D Systems	AF950	1:1000
nNOS	Santa Cruz	sc-5302	1:500
Tubulin	DSHB	12G10	1:2000
GAPDH	Santa Cruz	Sc-51907	1:1000
